# Supplementary material for: Analysis of subunit folding contribution of three yeast large ribosomal subunit proteins required for stabilisation and processing of intermediate nuclear rRNA precursors
Source: PLoS One. 2021 Nov 23;16(11):e0252497. doi: 10.1371/journal.pone.0252497 (PMC8610266; doi:10.1371/journal.pone.0252497)

A) Mature LSU, r-protein clusters not collapsed

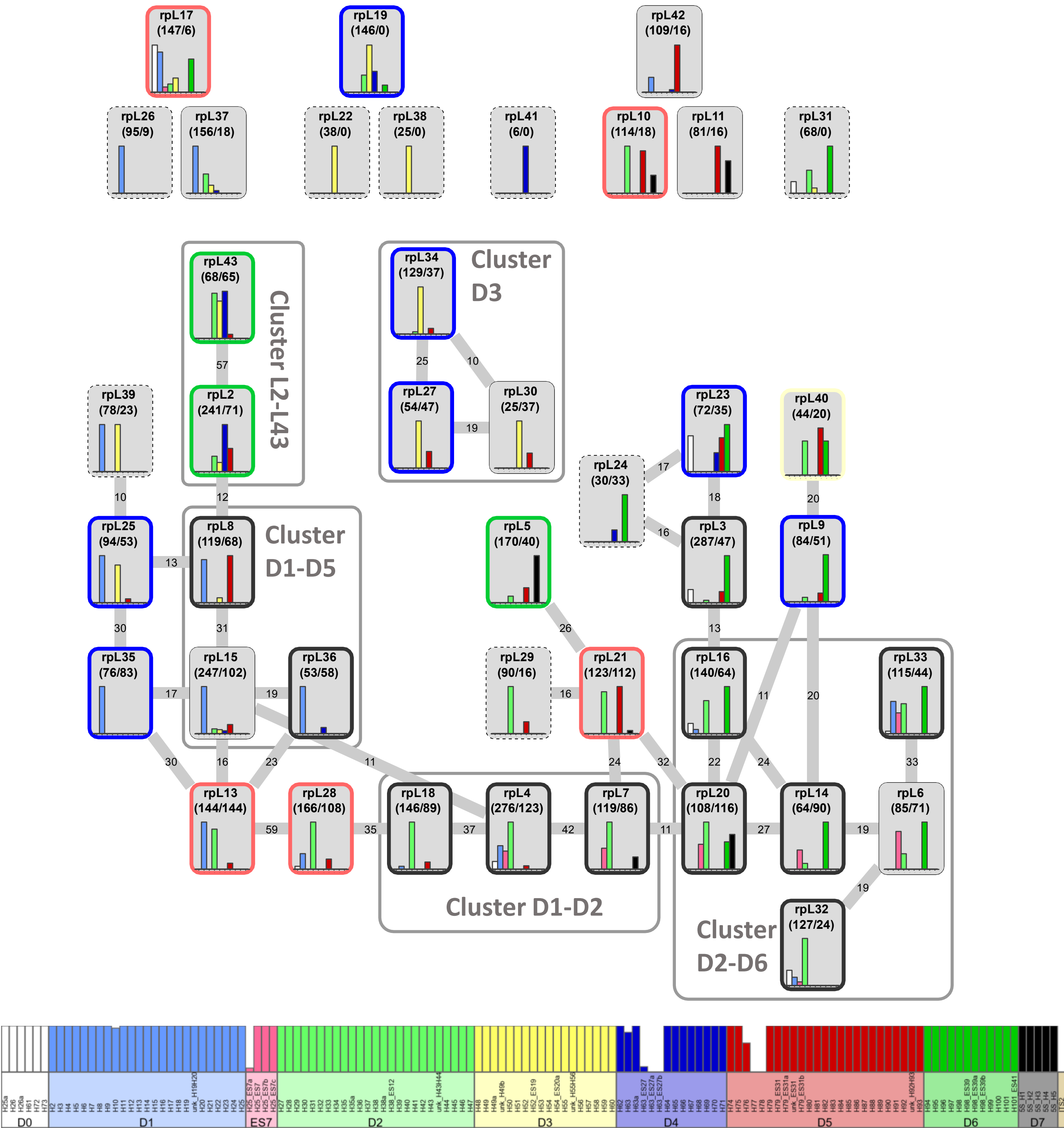

B) Mature LSU, r-protein clusters collapsed

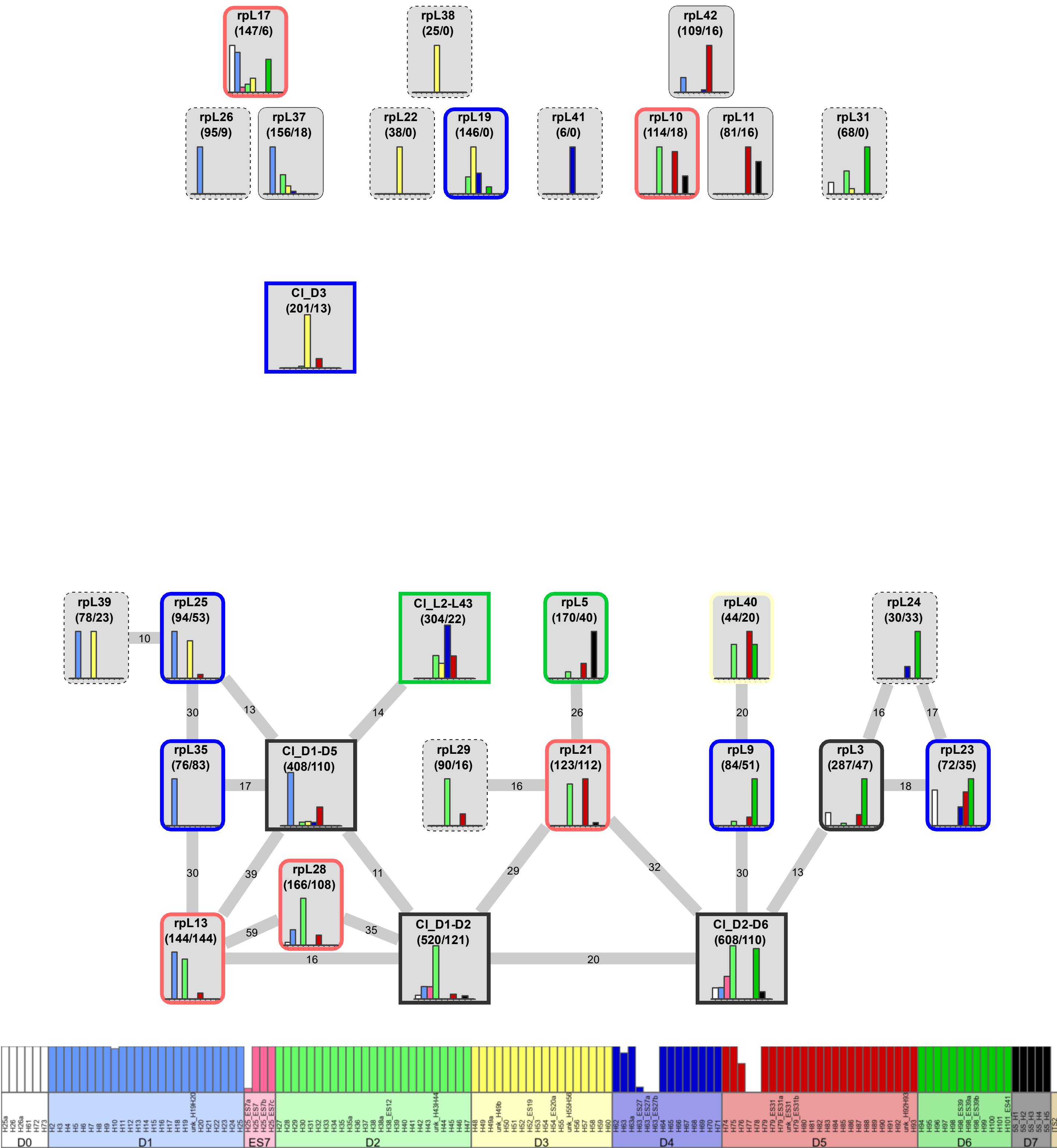

C) Nog1TAP-A

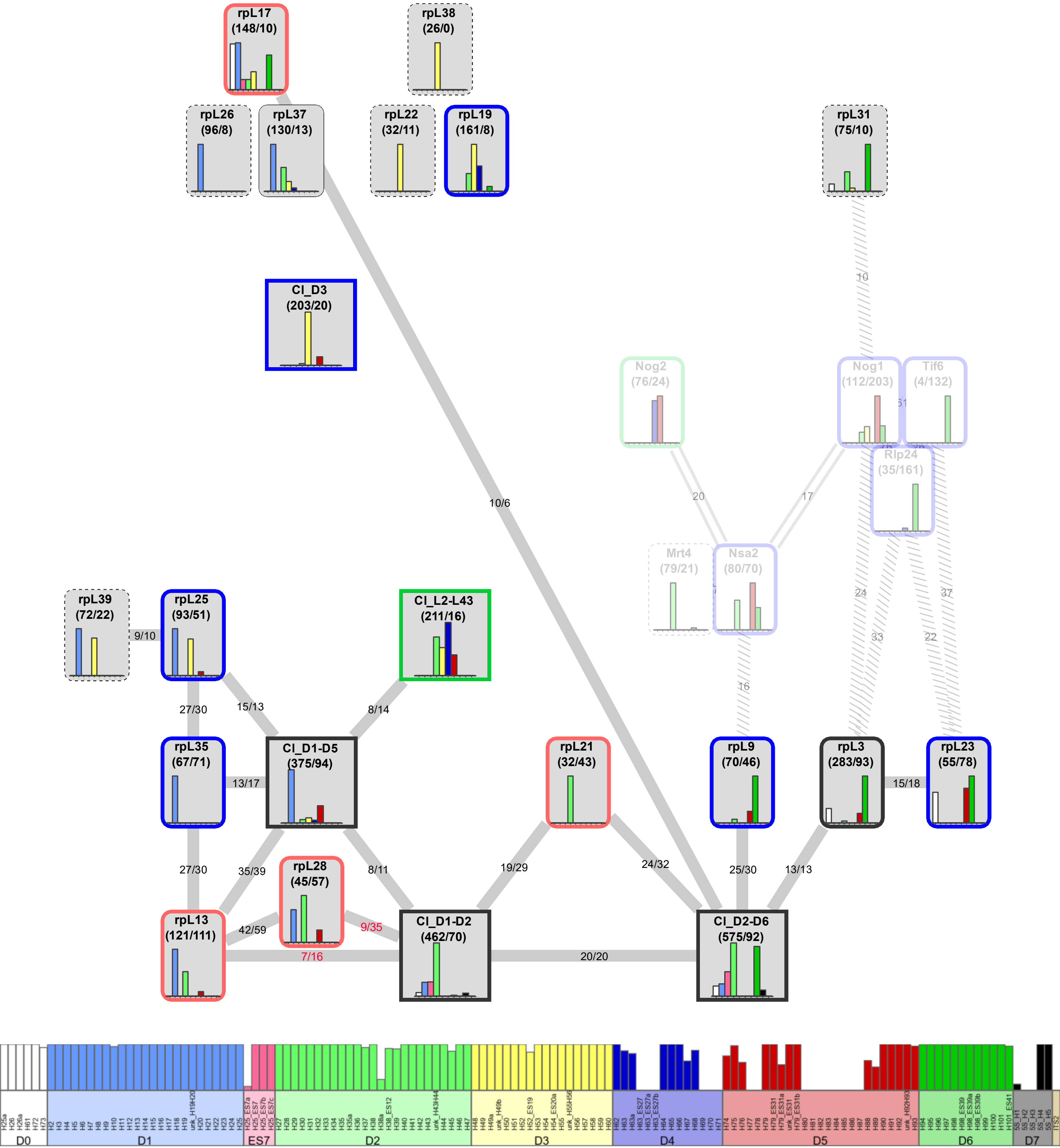
rpL17
(148/10)
rpL38
(26/0)
rpL26
(96/8)
rpL37
(130/13)
rpL22
(32/11)
rpL19
(161/8)
rpL31
(75/10)
10
Cl_D3
(203/20)
Nog2
(76/24)
Nog1
(112/203)
Tif6
(4/132)
Rlp24
(35/161)
10/6
20
17
Mrt4
(79/21)
Nsa2
(80/70)
24
37
33
22
16
rpL39
(72/22)
9/10
rpL25
(93/51)
Cl_L2-L43
(211/16)
27/30
15/13
8/14
rpL35
(67/71)
13/17
Cl_D1-D5
(375/94)
rpL21
(32/43)
rpL9
(70/46)
rpL3
(283/93)
15/18
rpL23
(55/78)
27/30
35/39
rpL28
(45/57)
8/11
19/29
24/32
25/30
13/13
rpL13
(121/111)
42/59
9/35
7/16
Cl_D1-D2
(462/70)
20/20
Cl_D2-D6
(575/92)
D0
D1
ES7
D2
D3
D4
D5
D6
D7

D) Nog1TAP-B

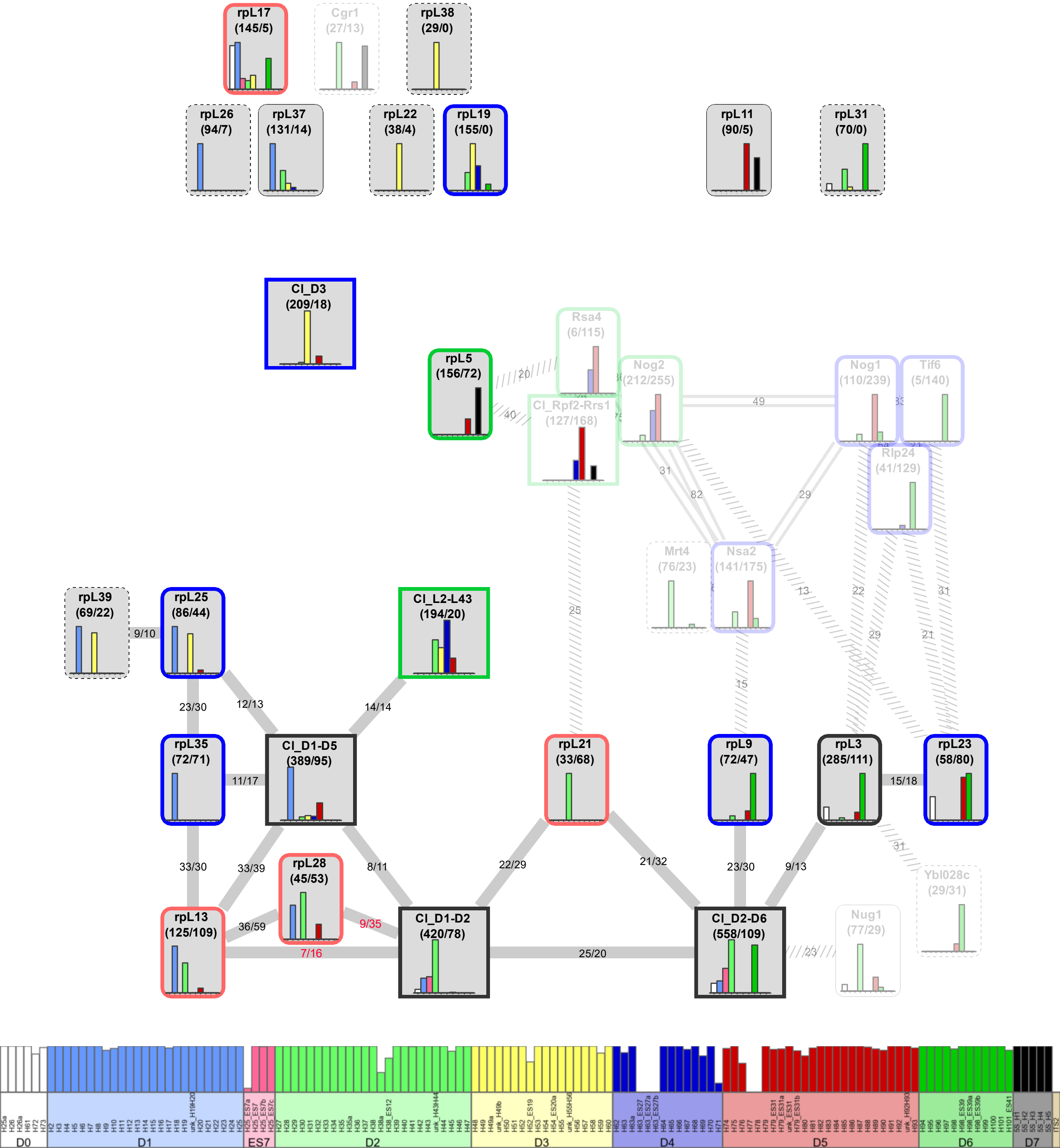

rpL17
(145/5)
Cgr1
(27/13)
rpL38
(29/0)
rpL26
(94/7)
rpL37
(131/14)
rpL22
(38/4)
rpL19
(155/0)
rpL11
(90/5)
rpL31
(70/0)
CI_D3
(209/18)
rpL5
(156/72)
Rsa4
(6/115)
Nog2
(212/255)
Nog1
(110/239)
Tif6
(5/140)
CI_Rpf2-Rrs1
(127/168)
Rlp24
(41/129)
Mrt4
(76/23)
Nsa2
(141/175)
rpL39
(69/22)
rpL25
(86/44)
CI_L2-L43
(194/20)
9/10
23/30
12/13
14/14
20
40
49
31
82
29
25
13
22
31
29
21
15
rpL35
(72/71)
CI_D1-D5
(389/95)
rpL21
(33/68)
rpL9
(72/47)
rpL3
(285/111)
rpL23
(58/80)
11/17
15/18
33/30
33/39
8/11
22/29
21/32
23/30
9/13
31
rpL28
(45/53)
Ybl028c
(29/31)
rpL13
(125/109)
CI_D1-D2
(420/78)
CI_D2-D6
(558/109)
Nug1
(77/29)
36/59
9/35
7/16
25/20
23
D0
D1
ES7
D2
D3
D4
D5
D6
D7

E) Nog1TAP-C

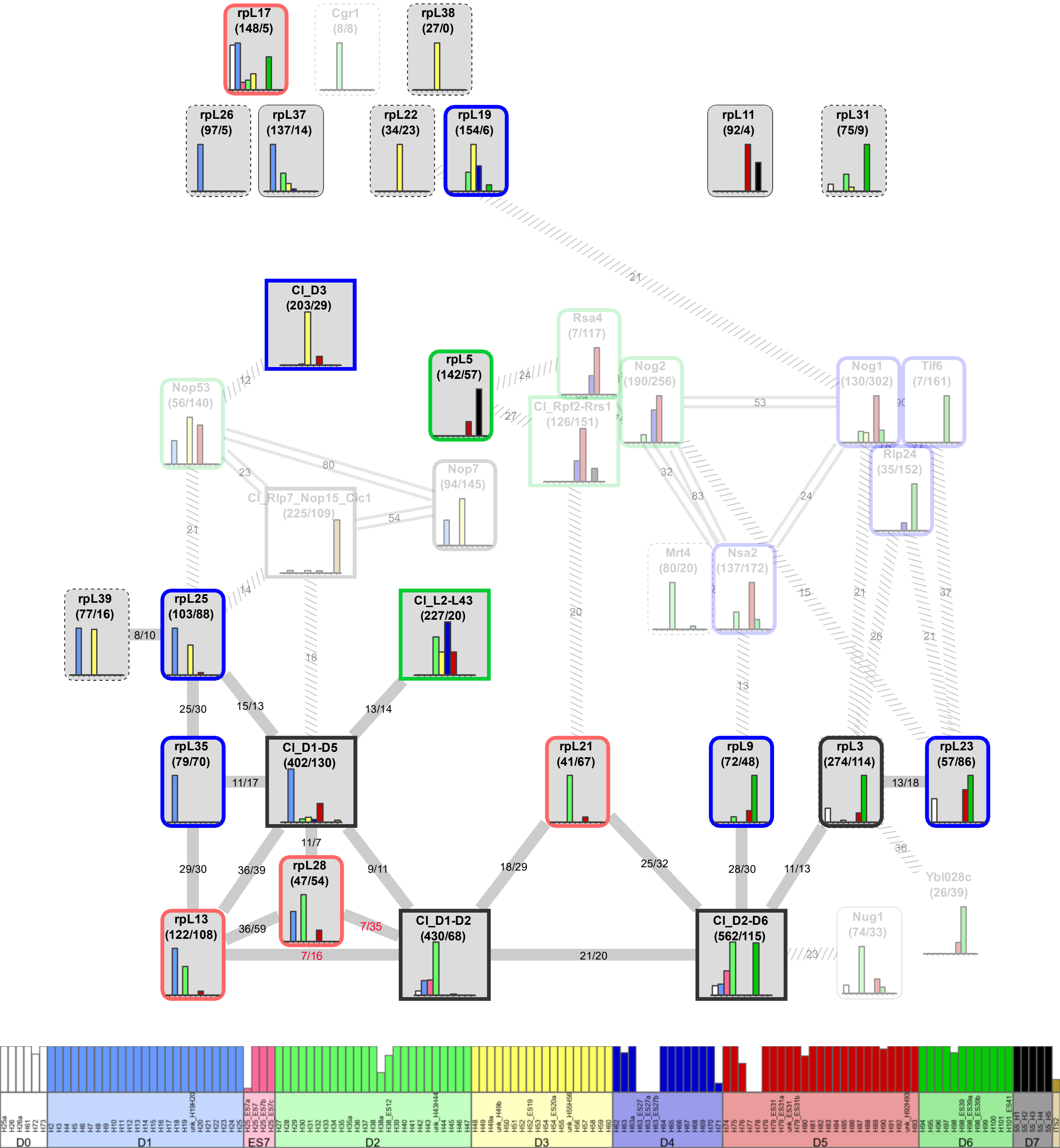
rpL17 (148/5)
Cgr1 (8/8)
rpL38 (27/0)
rpL26 (97/5)
rpL37 (137/14)
rpL22 (34/23)
rpL19 (154/6)
rpL11 (92/4)
rpL31 (75/9)
21
Cl_D3 (203/29)
Rsa4 (7/117)
rpL5 (142/57)
Nog2 (190/256)
Nog1 (130/302)
Tif6 (7/161)
Nop53 (56/140)
12
24
27
Cl_Rpf2-Rrs1 (126/151)
53
Nop7 (94/145)
80
23
Cl_Rlp7_Nop15_Cic1 (225/109)
54
32
83
24
Rlp24 (35/152)
21
Mrt4 (80/20)
Nsa2 (137/172)
rpL39 (77/16)
rpL25 (103/88)
8/10
14
Cl_L2-L43 (227/20)
20
18
15
21
37
26
21
13
25/30
15/13
13/14
rpL35 (79/70)
Cl_D1-D5 (402/130)
11/17
rpL21 (41/67)
rpL9 (72/48)
rpL3 (274/114)
rpL23 (57/86)
13/18
11/7
36
29/30
36/39
rpL28 (47/54)
9/11
18/29
25/32
28/30
11/13
Ybl028c (26/39)
rpL13 (122/108)
36/59
7/35
Cl_D1-D2 (430/68)
Cl_D2-D6 (562/115)
Nug1 (74/33)
7/16
21/20
23
D0
D1
ES7
D2
D3
D4
D5
D6
D7

F) Nog1TAP-E

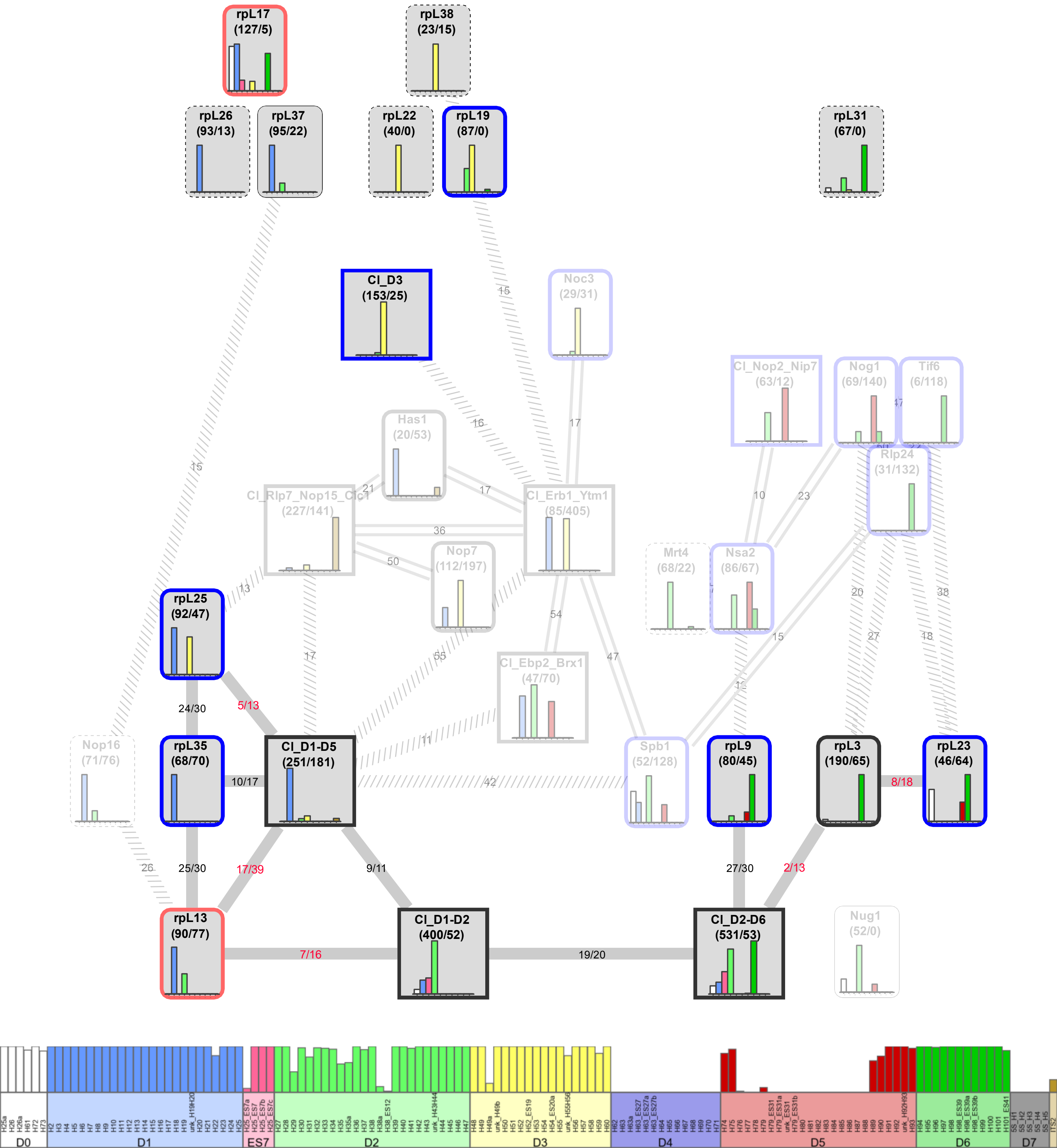

rpL17 (127/5)
rpL38 (23/15)
rpL26 (93/13)
rpL37 (95/22)
rpL22 (40/0)
rpL19 (87/0)
rpL31 (67/0)
Cl_D3 (153/25)
Noc3 (29/31)
Cl_Nop2_Nip7 (63/12)
Nog1 (69/140)
Tif6 (6/118)
Has1 (20/53)
Rlp24 (31/132)
Cl_Rlp7_Nop15_Cic1 (227/141)
Cl_Erb1_Ytm1 (85/405)
Nop7 (112/197)
Mrt4 (68/22)
Nsa2 (86/67)
rpL25 (92/47)
Cl_Ebp2_Brx1 (47/70)
Nop16 (71/76)
rpL35 (68/70)
Cl_D1-D5 (251/181)
Spb1 (52/128)
rpL9 (80/45)
rpL3 (190/65)
rpL23 (46/64)
rpL13 (90/77)
Cl_D1-D2 (400/52)
Cl_D2-D6 (531/53)
Nug1 (52/0)
15
15
16
17
17
21
17
36
50
10
23
13
54
17
55
47
20
38
27
18
15
24/30
5/13
11
10/17
42
8/18
26
25/30
17/39
9/11
27/30
2/13
7/16
19/20
D0
D1
ES7
D2
D3
D4
D5
D6
D7

G) Nog1TAP-F

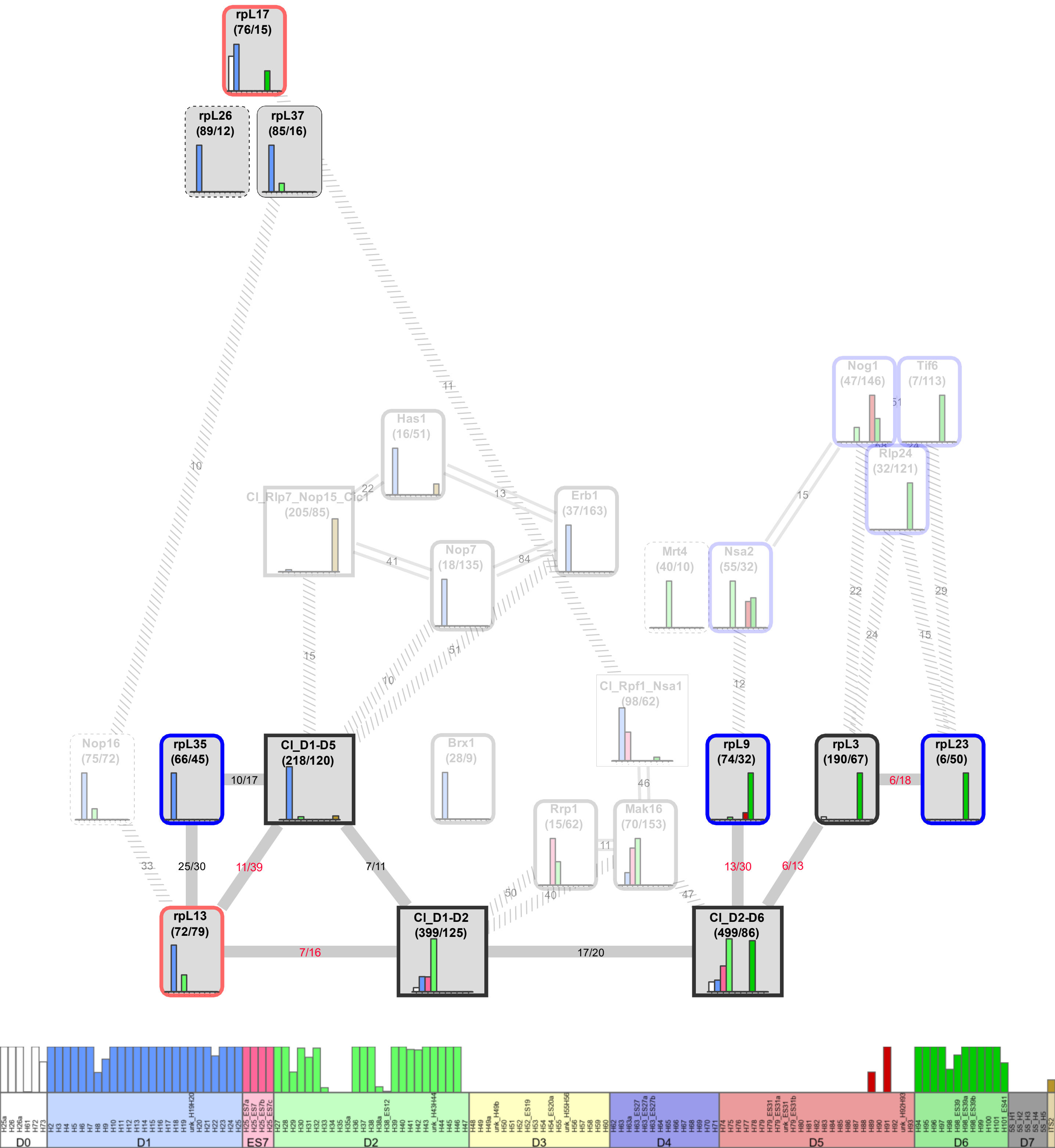
rpL17
(76/15)
rpL26
(89/12)
rpL37
(85/16)
Nog1
(47/146)
Tif6
(7/113)
Rlp24
(32/121)
Has1
(16/51)
Cl_Rlp7_Nop15_Cic1
(205/85)
Erb1
(37/163)
Nop7
(18/135)
Mrt4
(40/10)
Nsa2
(55/32)
Cl_Rpf1_Nsa1
(98/62)
Nop16
(75/72)
rpL35
(66/45)
Cl_D1-D5
(218/120)
Brx1
(28/9)
rpL9
(74/32)
rpL3
(190/67)
rpL23
(6/50)
Rrp1
(15/62)
Mak16
(70/153)
rpL13
(72/79)
Cl_D1-D2
(399/125)
Cl_D2-D6
(499/86)
10/17
25/30
11/39
7/11
13/30
6/13
6/18
7/16
17/20
D0
D1
ES7
D2
D3
D4
D5
D6
D7

H) Nog1TAP_L2-A

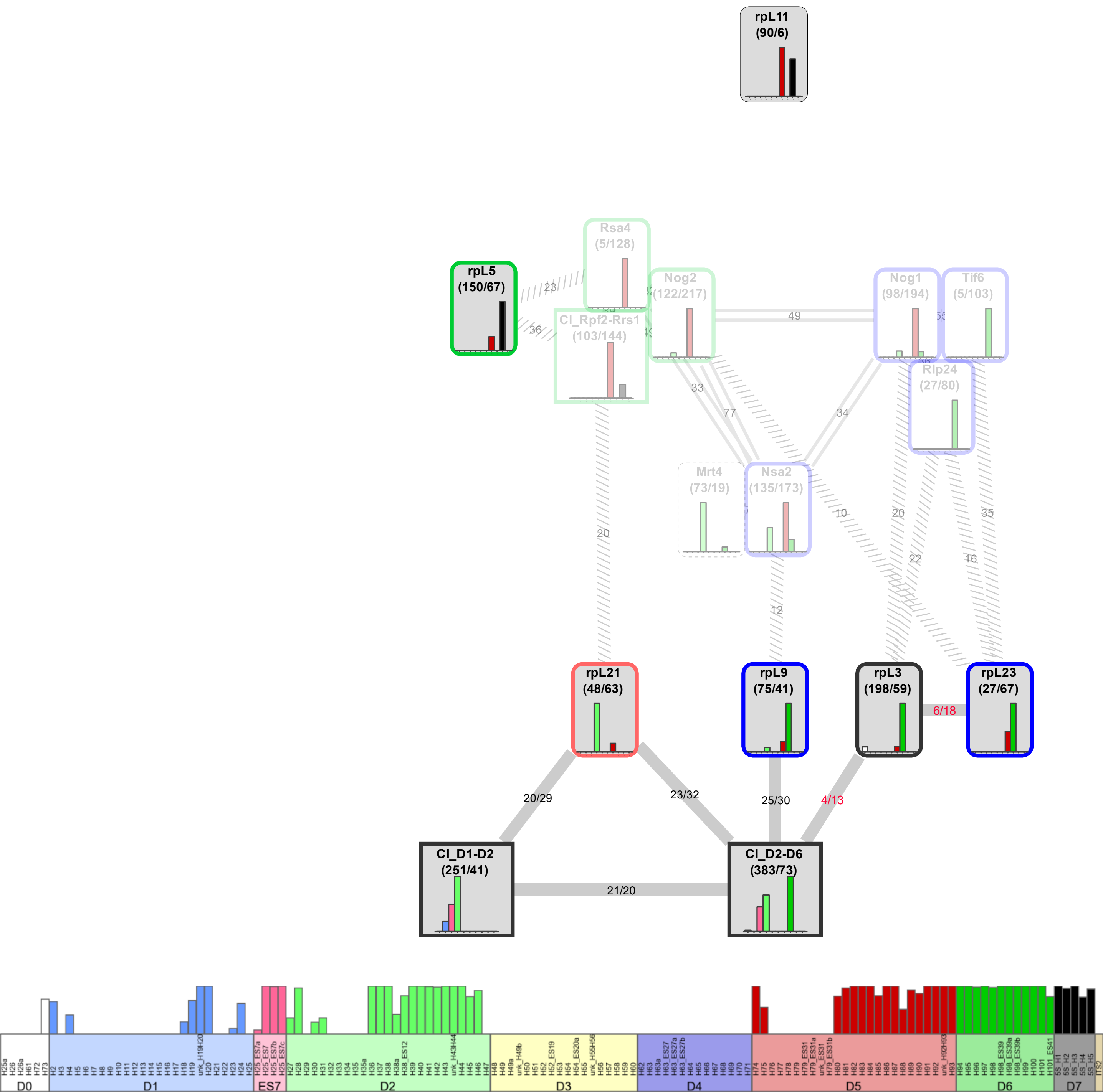

I) Nog1TAP_L2-B

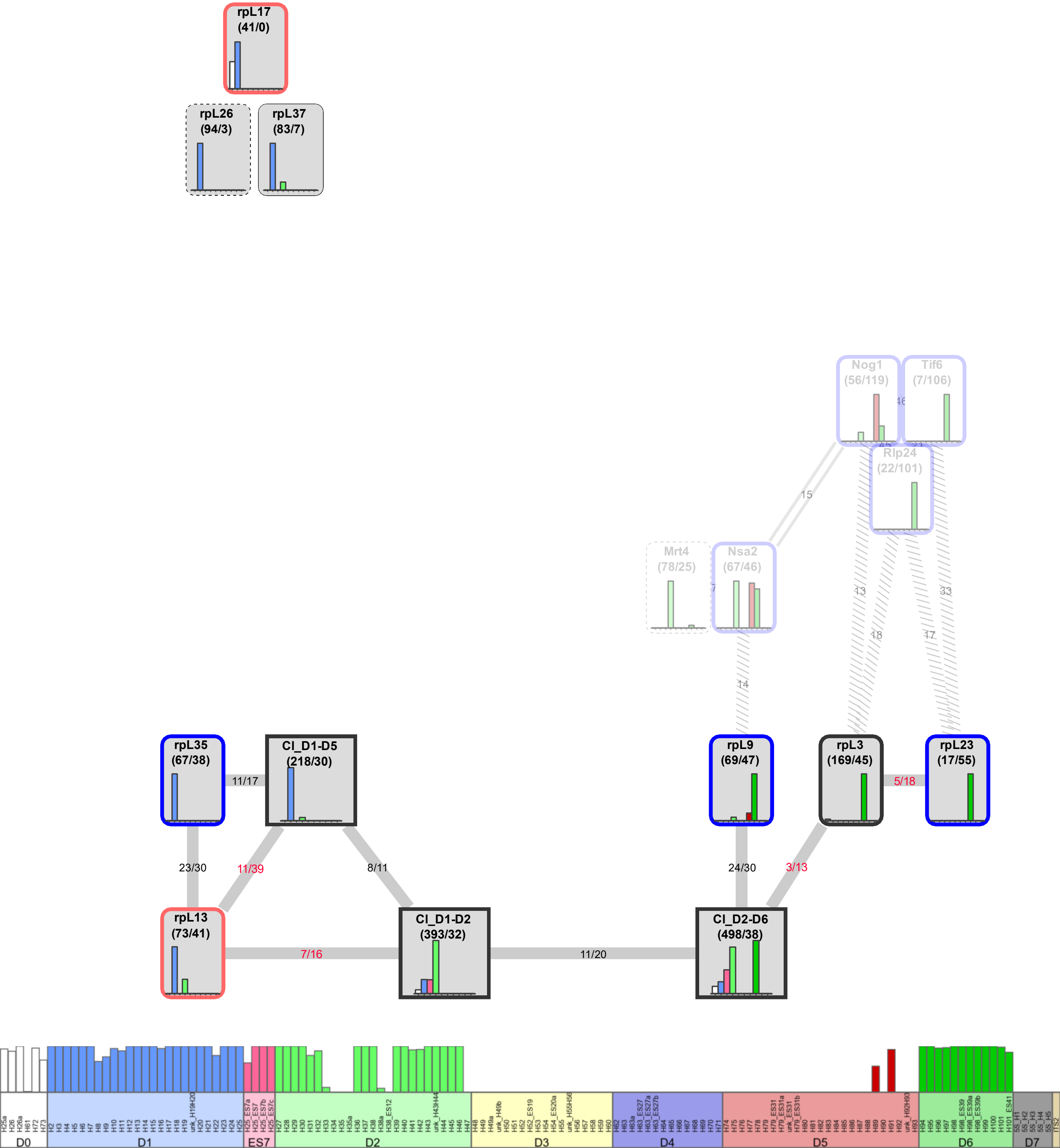

J) Nog1TAP_L2-C

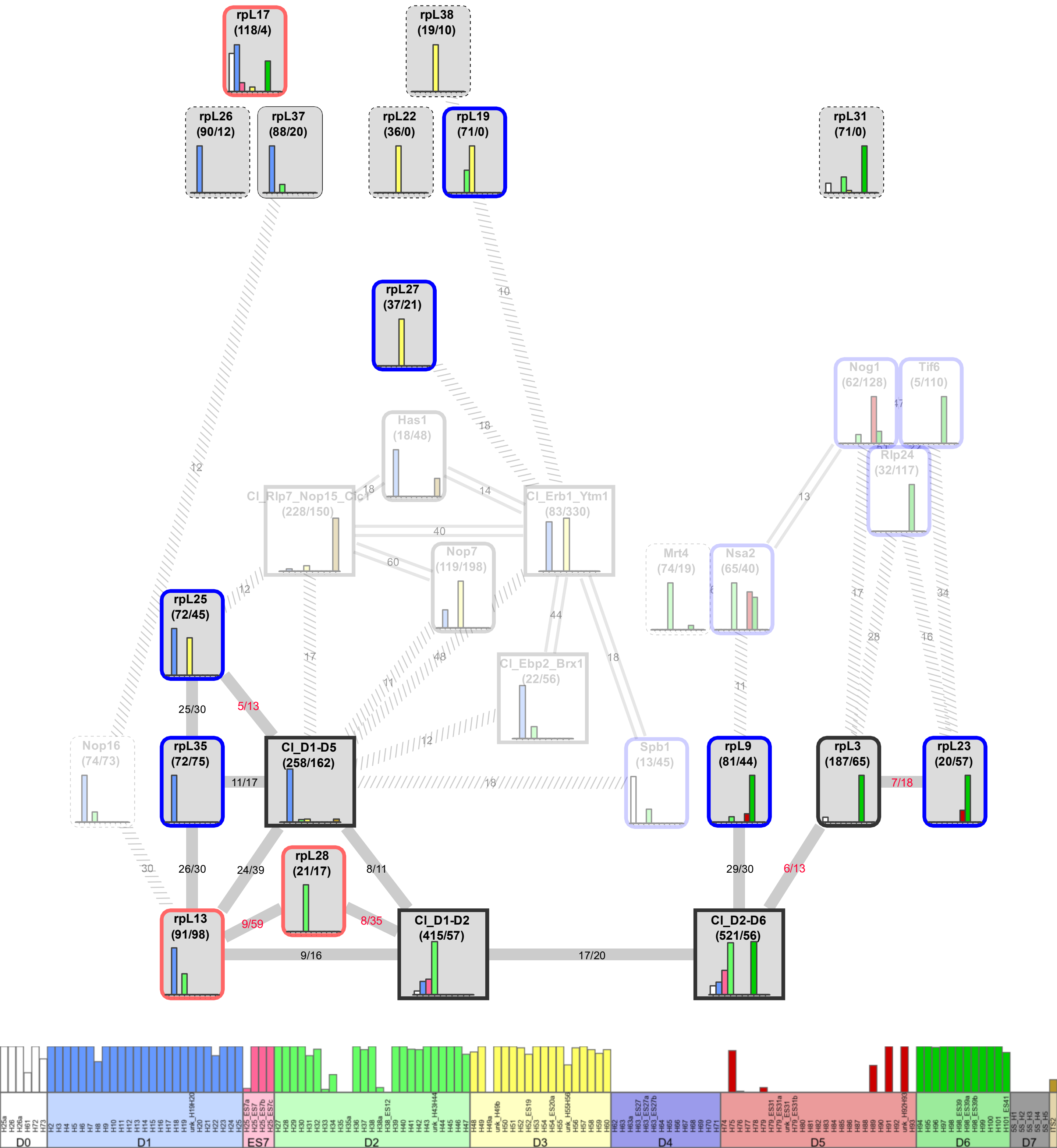

K) Nog1TAP_L25-A

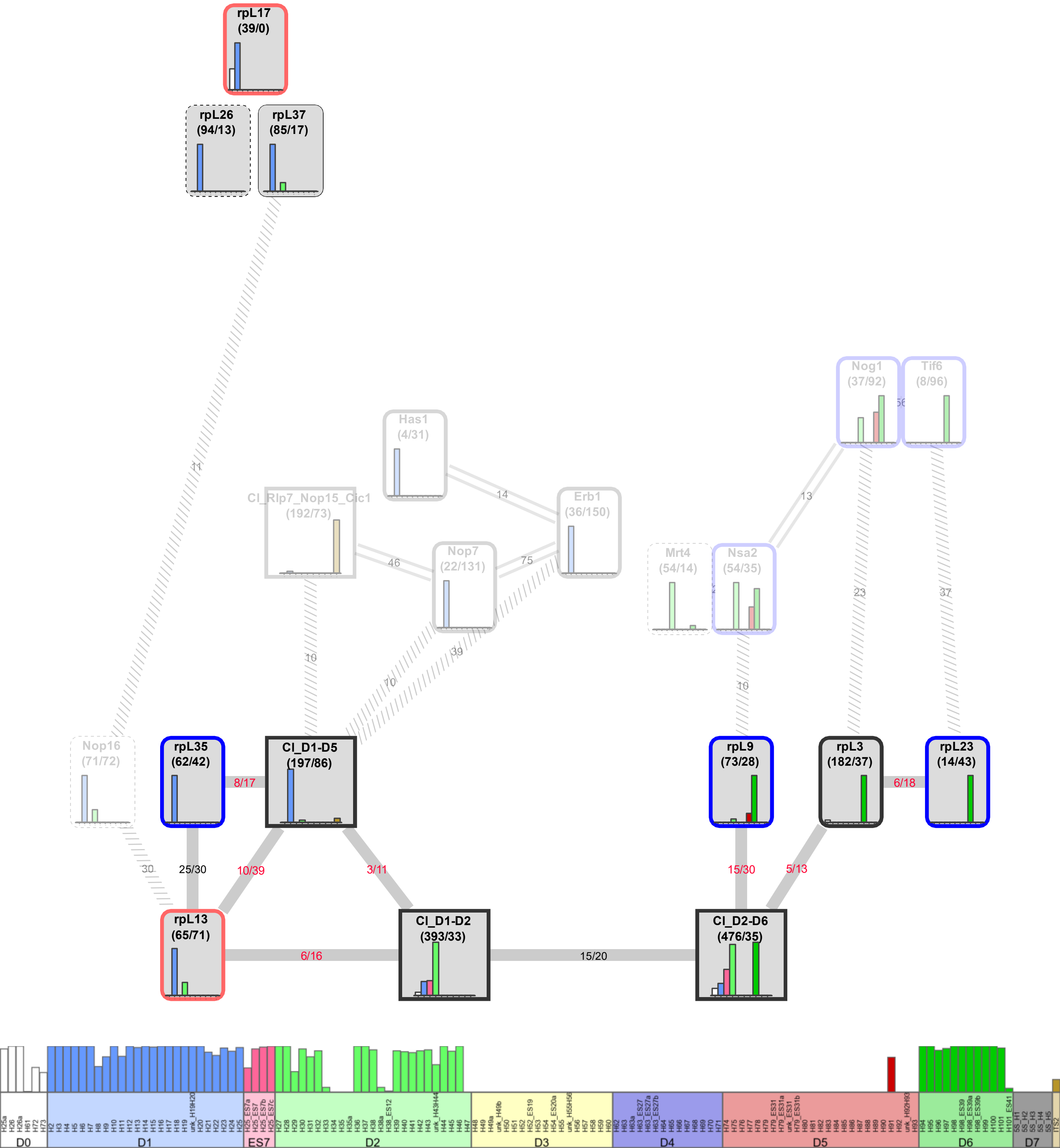

L) Nog1TAP_L25-B

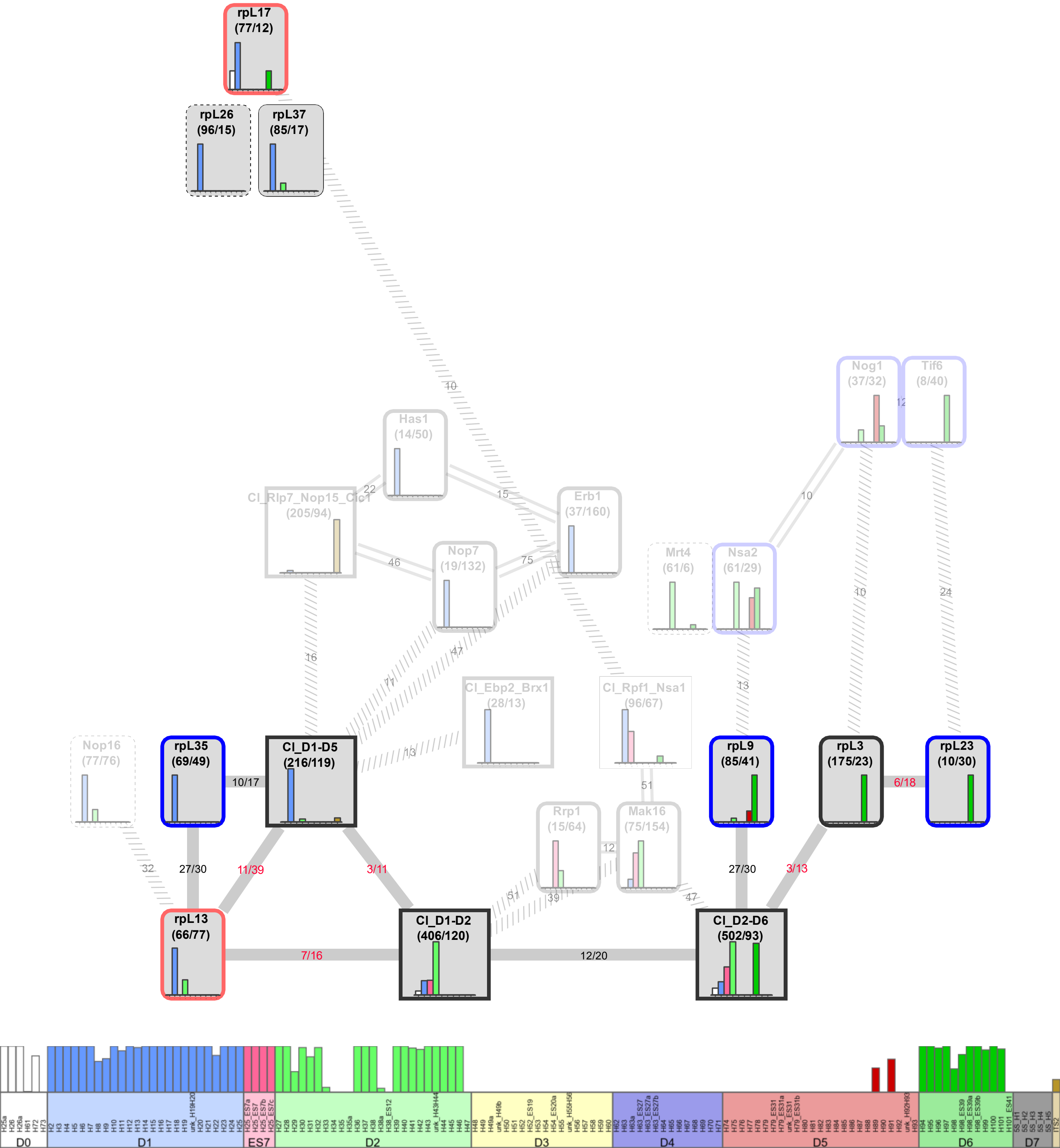

M) Nog1TAP_L34-A

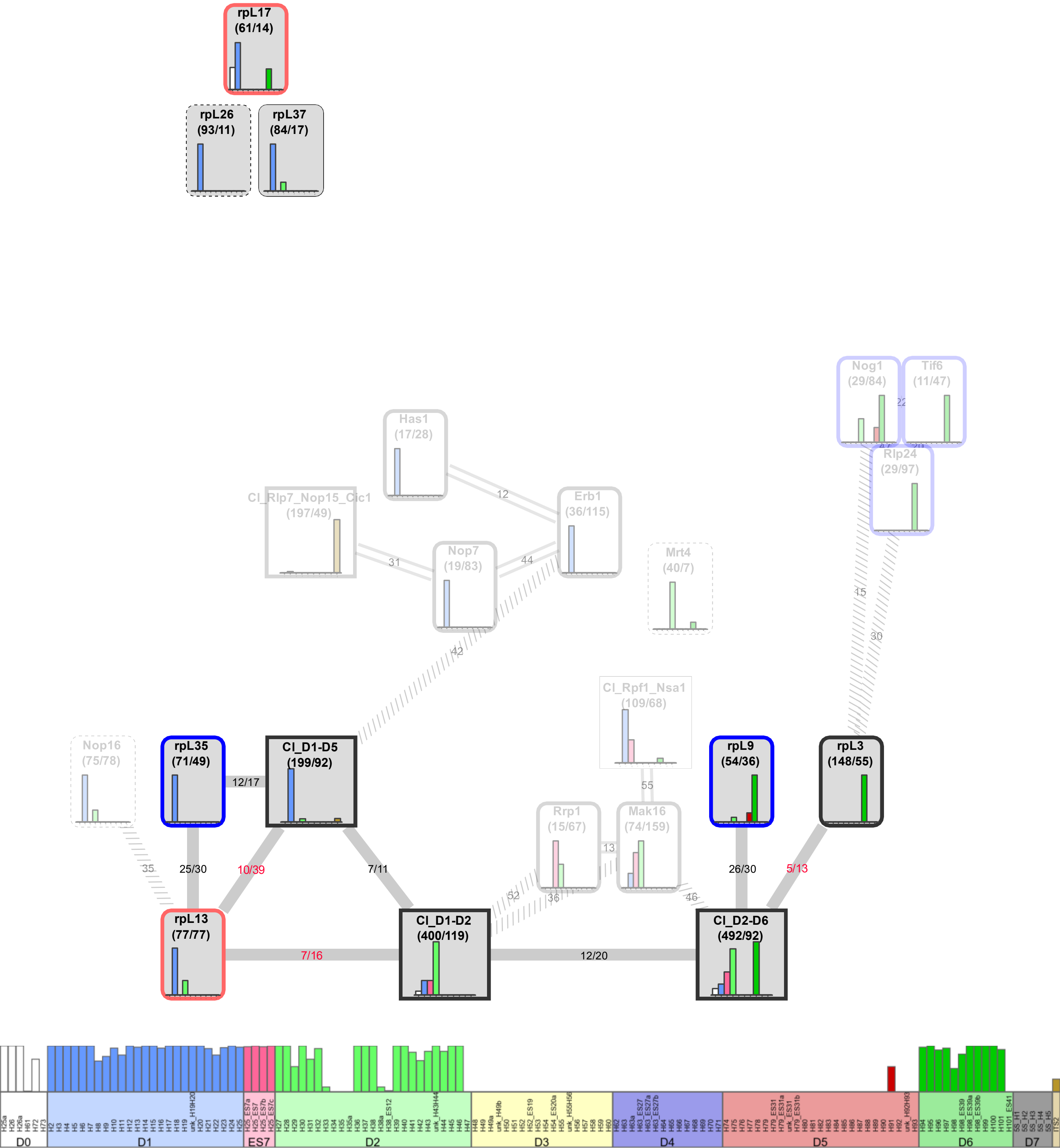

N) Nog1TAP_L34-B

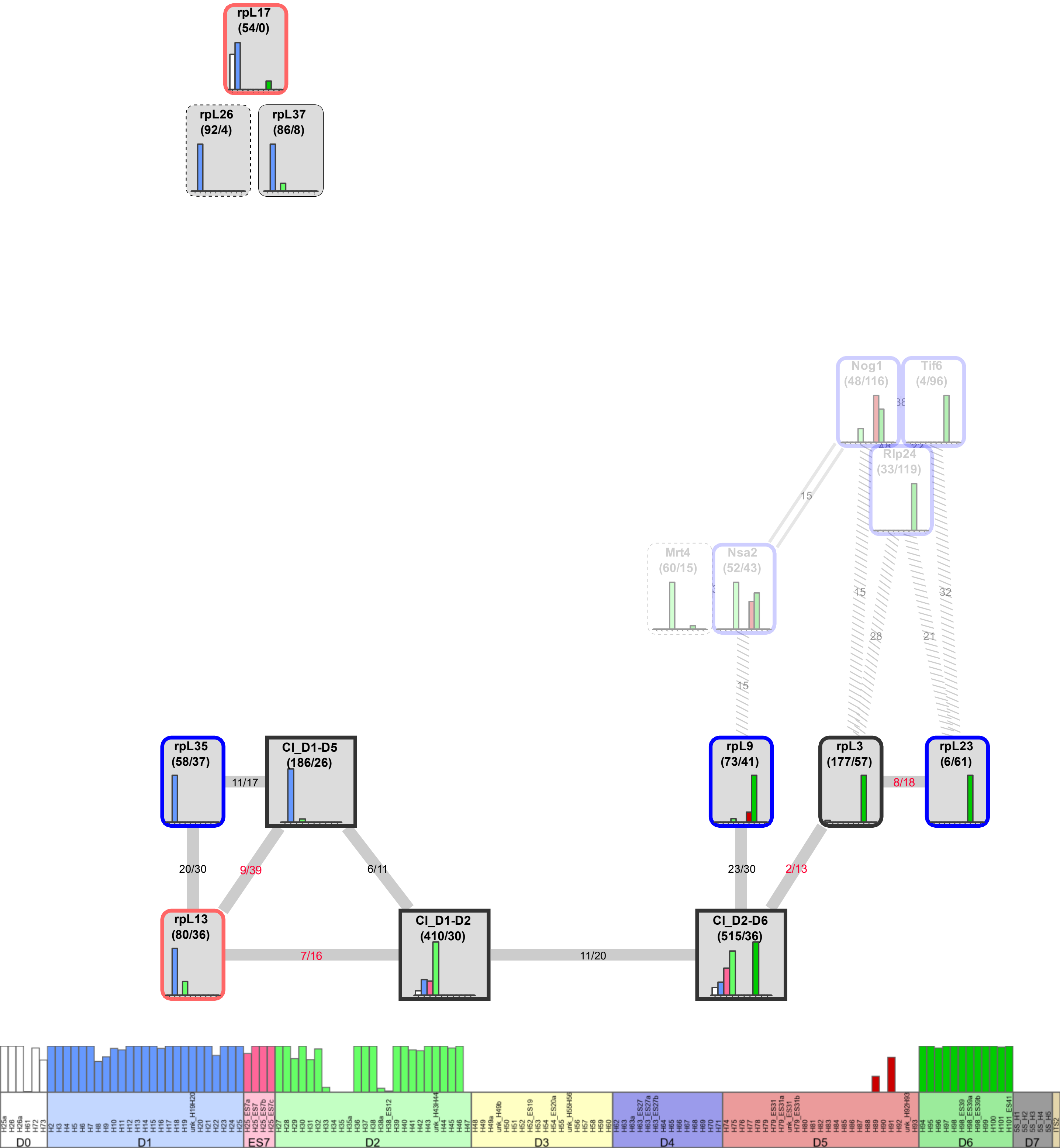

Supplement: S1 Appendix — Individual proteins are represented in (A)–(N) by rounded corners, groups of proteins by rectangles with sharp corners. For space reasons throughout the Figure rRNA domains are indicated with Arabic numbering (D0, D1, D2, D3…). Definition of multi-component r-protein clusters designated Cl_D1-D5, Cl_D1-D2, Cl_D2-D6 and Cluster Cl_D3 and Cl_L2-L43 is shown in (A) and is based on protein—protein interactions and functional categories. These are symbolized in (A)–(N) for all proteins by the colour of the border of the rectangles: Proteins required for early pre-rRNA processing are represented by black bordered rectangles, for intermediate nuclear pre-rRNA processing steps by blue, for late nuclear pre-rRNA processing steps by green and for downstream nuclear maturation and export by red bordered rectangles. Boxes for proteins with unclear function and non-essential proteins have grey borders, with the latter ones in dotted lines (see also S6 Appendix for definition of functional categories). Interactions between individual proteins, groups of proteins and rRNA domains were deduced from residue—residue proximities in the structure models (see Materials and Methods). Predicted major interaction interfaces with > = 10 residues involved (respective numbers are shown, sums of residues in both partners) are visualized between r-protein (groups) by grey lines, between (groups of) factors by white lines and between factors and r-proteins by interrupted lines. For r-protein interactions with r-proteins also the ones involving less than 10 residues are visualized and the number behind the slash is the one observed in the mature LSU structure model 4u3u. Numbers falling below 50% of the ones deduced for pdb-databank entry 4u3u are highlighted in red. Predicted interactions of proteins with rRNA domains are shown as bar diagrams inside protein boxes (relative amounts). Colour code and order of rRNA domains in these diagrams are reflected in the bar diagram at the bottom of each fi [file pone.0252497.s001.pdf]
