# Supplementary figures and images for: Analysis of subunit folding contribution of three yeast large ribosomal subunit proteins required for stabilisation and processing of intermediate nuclear rRNA precursors
Source: PLoS One. 2021 Nov 23;16(11):e0252497. doi: 10.1371/journal.pone.0252497 (PMC8610266; doi:10.1371/journal.pone.0252497)

RPL controlled  
by pGAL1/10

Nog1-TAP

|   |    |       |
|---|----|-------|
| 1 | -- | --    |
| 2 | x  | --    |
| 3 | x  | RPL25 |
| 4 | x  | RPL2  |
| 5 | x  | RPL34 |

YPG

YPD

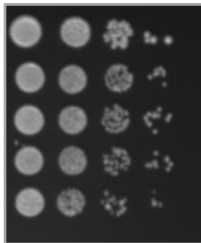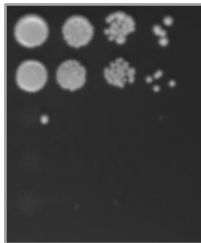

Supplement: S2 Appendix — Yeast strains BY4742 (lane 1), Y1877 (lane 2), Y1816 (lane 3), Y1921 (lane 4) and Y2907 (lane 5) were cultivated in galactose containing liquid full medium at 30°C and serial dilutions were then spotted on galactose (YPG) or glucose containing (YPD) solid medium. Images were taken after 72h incubation at 30°C. (PDF) [file pone.0252497.s002.pdf]

A

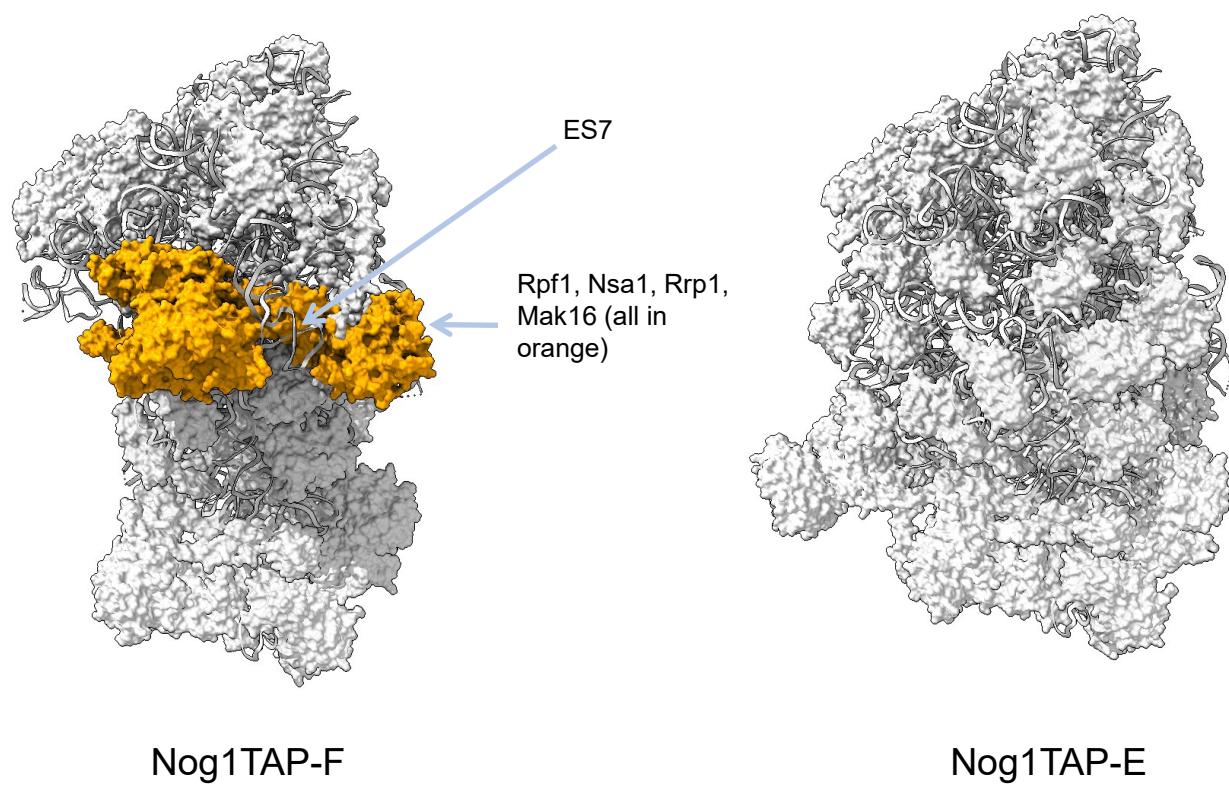

B

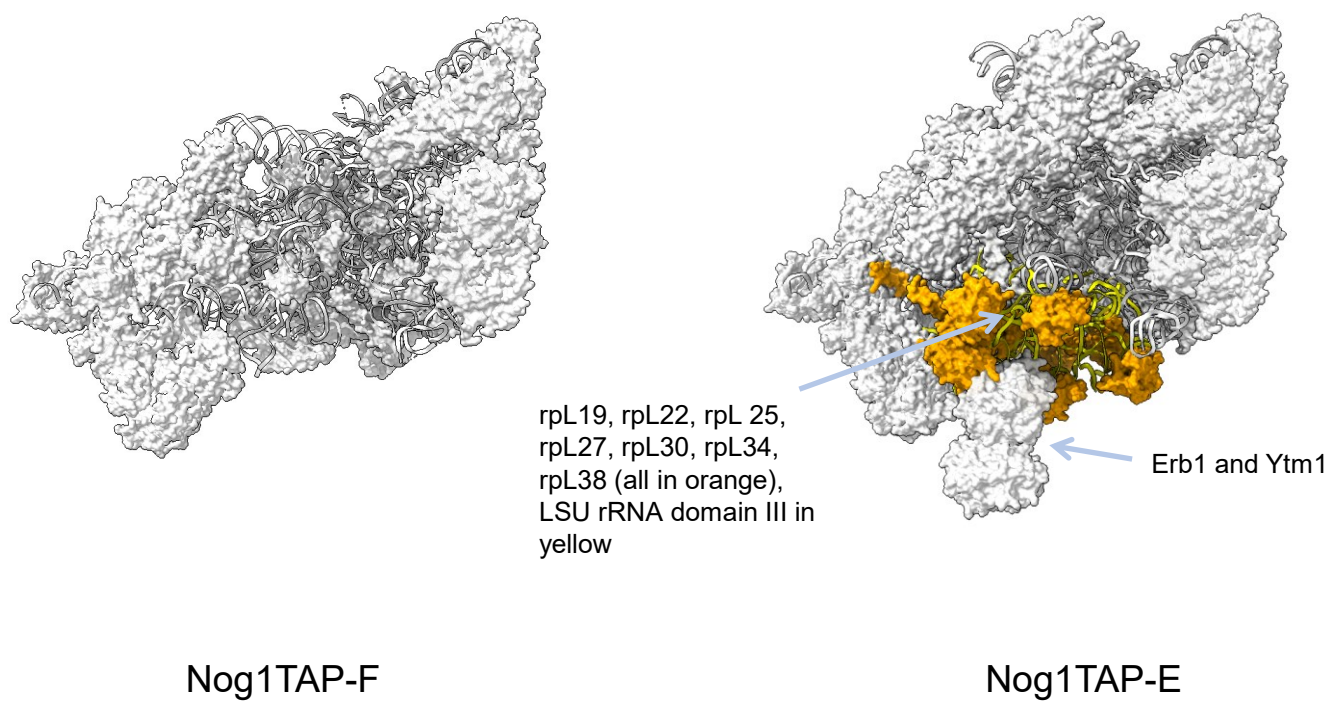

C

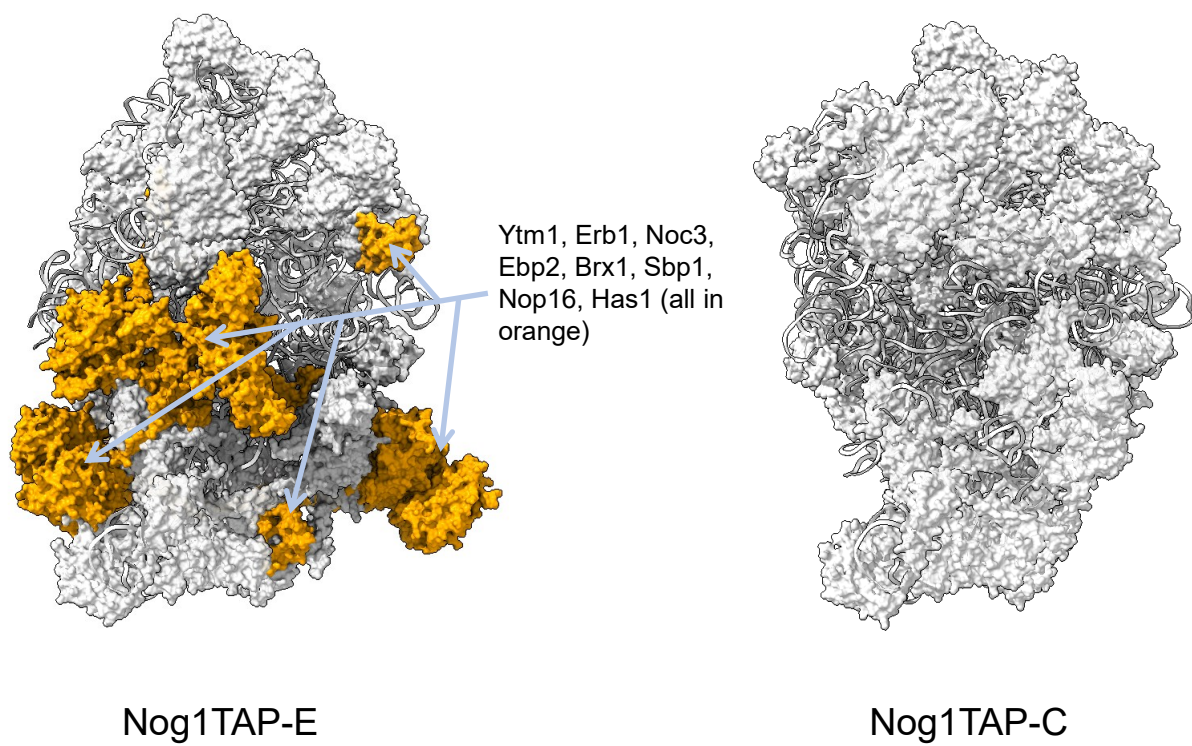

D

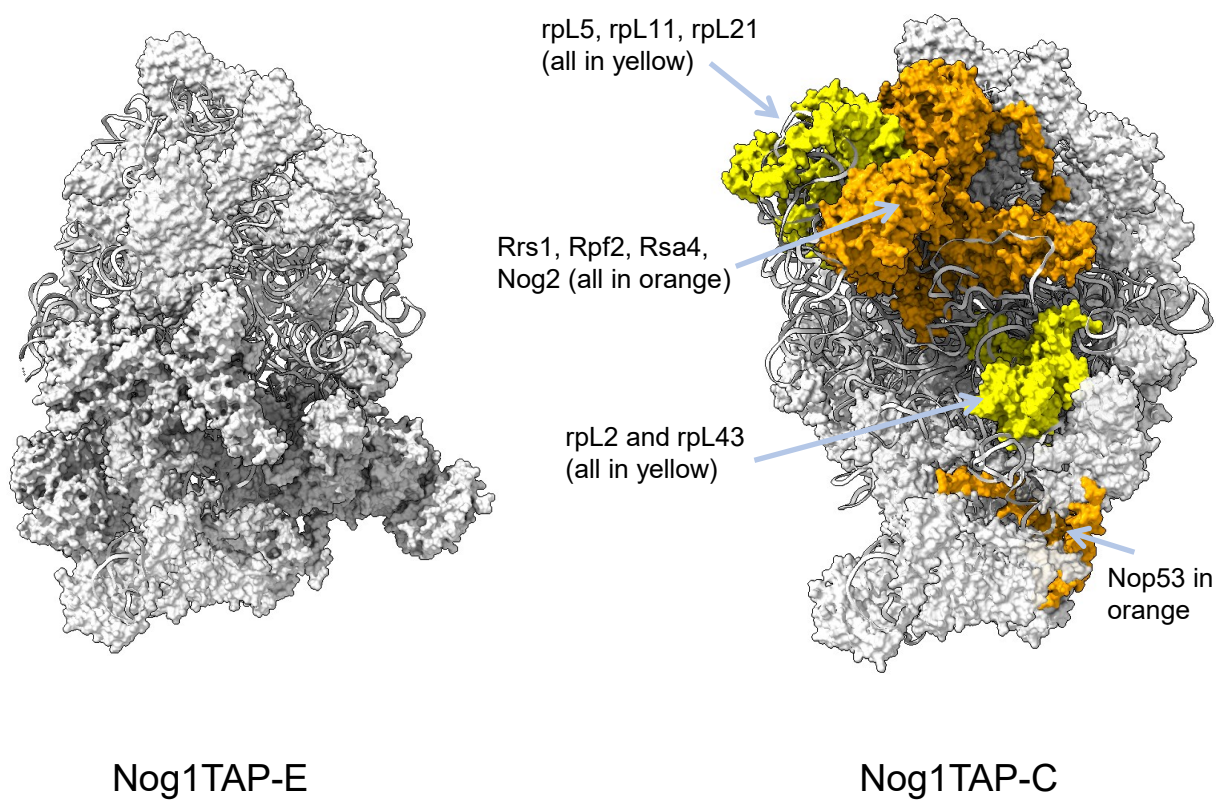

E

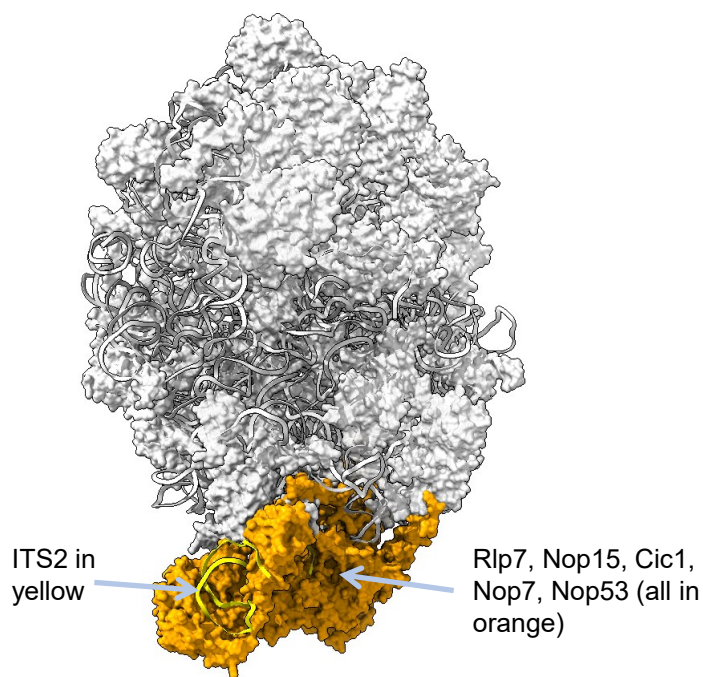

Nog1TAP-C

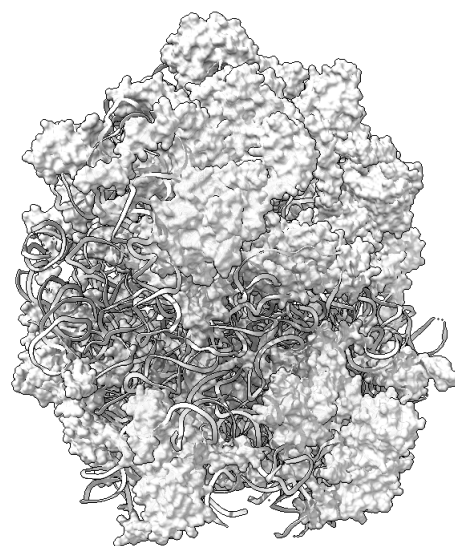

Nog1TAP-B

F

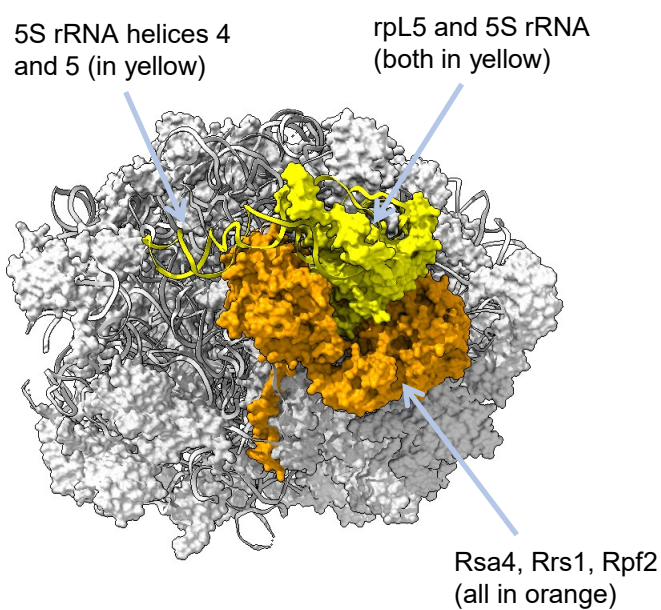

Nog1TAP-B

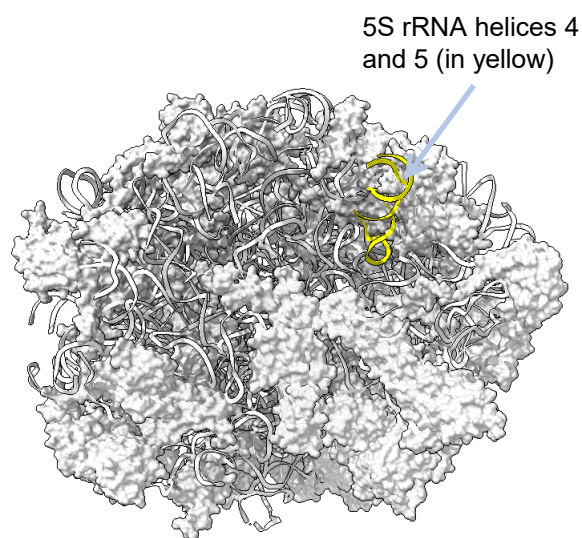

Nog1TAP-A

G

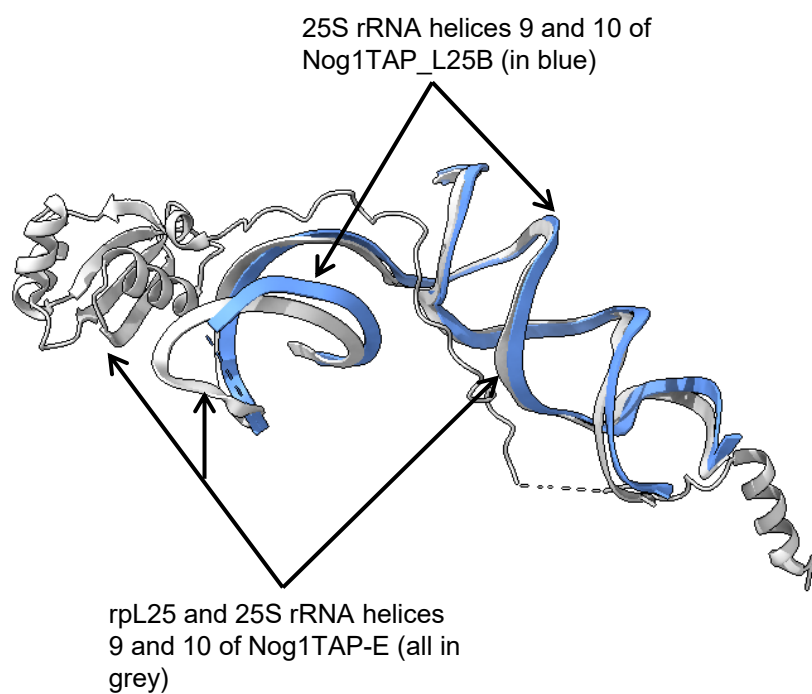

H

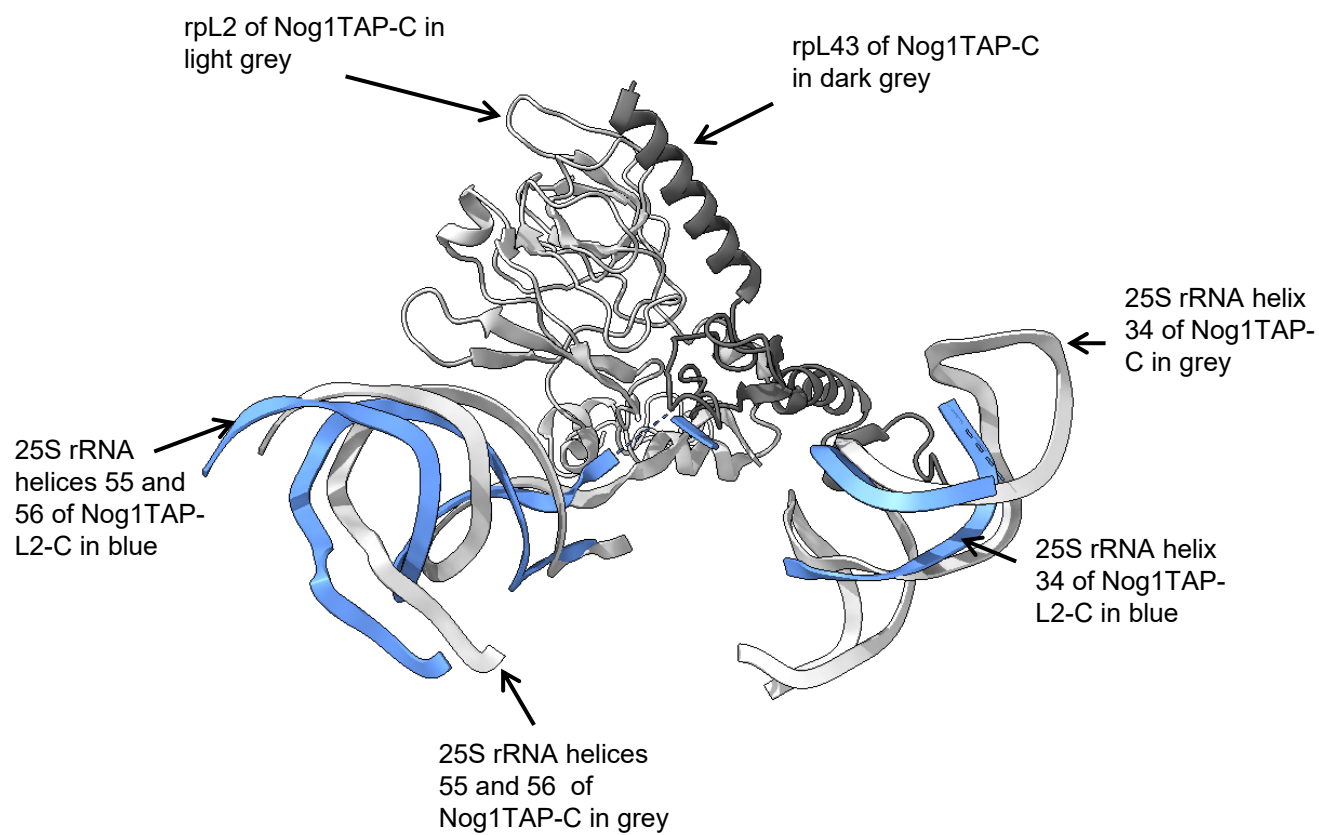

Supplement: S9 Appendix — The indicated structure models were visualized in ChimeraX with RNA shown in cartoon backbone representation and proteins in surface representation, except for G) and H) where proteins are also shown in cartoon backbone representation. In G) and H) models obtained from r-protein expression mutant strains were aligned by the matchmaker algorithm in ChimeraX to the indicated reference models based on the 25S rRNA chains. (PDF) [file pone.0252497.s009.pdf]
