# Supplementary material for: Analysis of subunit folding contribution of three yeast large ribosomal subunit proteins required for stabilisation and processing of intermediate nuclear rRNA precursors
Source: PLoS One. 2021 Nov 23;16(11):e0252497. doi: 10.1371/journal.pone.0252497 (PMC8610266; doi:10.1371/journal.pone.0252497)
Supplement: S3 Appendix — Map resolution estimates (half maps, fourier shell correlation threshold 0.143) are given as reported by relion (user-created mask) and by phenix validation tools (no user-created mask). Model statistics as reported by phenix validation tools and accession numbers for models (wwPDB), related density maps (EMDB) and for full EM-datasets (EMPIAR) are indicated. Fourier shell correlation (FSC) graphs as reported by phenix validation tools are shown in S4 Appendix. (PDF) [file pone.0252497.s003.pdf]

	Nog1TAP-A	Nog1TAP-B	Nog1TAP-C	Nog1TAP-E
Data collection				
Voltage (kV)	300	300	300	300
Pixel size (A)	1.06	1.06	1.06	1.06
Electron exposure (e(A^2)	84.67	84.67	84.67	84.67
Fractions	40	40	40	40
Exposure time	5.16	5.16	5.16	5.16
wwPDB accession code	7OF1	7OH3	7OHQ	7OHR
EMDB accession code	EMD-12866	EMD-12892	EMD-12905	EMD-12906
EMPIAR accession code	EMPIAR-10776	EMPIAR-10776	EMPIAR-10776	EMPIAR-10776
Map resolution estimates (half maps, FSC threshold 0.143)				
Relion (user mask)	3.1	3.4	3.1	4.7
Phenix (autom. mask)	3.1	3.6	3.1	4.8
Model composition				
starting model	6n8j	3jct	3jct	6elz
Chains	44	55	61	52
Protein Residues	6344	8308	9547	9318
Nucleotides	2988	3289	3397	2596
Bonds (RMSD)				
Length (A)	0.004	0.002	0.003	0.005
Angles (°)	0.638	0.657	0.693	0.700
Validation				
MolProbity score	1.60	1.74	1.85	1.58
Clash score	7.21	7.88	8.49	7.25
Ramachandran plot (%)				
Outliers	0.03	0.04	0.07	0.04
Allowed	3.21	4.38	5.70	3.04
Favored	96.76	95.58	94.23	96.91
Rotamer outliers (%)	0.77	0.68	0.84	0.99
Model vs. Data				
CC (mask)	0.67	0.76	0.75	0.65
CC (box)	0.79	0.84	0.82	0.76
CC (peaks)	0.64	0.72	0.69	0.57
CC (volume)	0.67	0.75	0.75	0.66

	Nog1TAP-F	Nog1TAP_L2-A	Nog1TAP_L2-B	Nog1TAP_L2-C
Data collection				
Voltage (kV)	300	300	300	300
Pixel size (A)	1.06	1.06	1.06	1.06
Electron exposure (e(A^2)	84.67	86.45	86.45	86.45
Fractions	40	40	40	40
Exposure time	5.16	5.16	5.16	5.16
wwPDB accession code	7OHS	7OHT	7OHU	7OHV
EMDB accession code	EMD-12907	EMD-12908	EMD-12909	EMD-12910
EMPIAR accession code	EMPIAR-10776	EMPIAR-10780	EMPIAR-10780	EMPIAR-10780
Map resolution estimates (half m				
Relion (user mask)	4.4	4.7	3.7	3.9
Phenix (autom. mask)	4.5	5.0	3.8	4.0
Model composition				
starting model	6em1	3jct	6em1	6elz
Chains	42	30	27	47
Protein Residues	6971	5154	4202	7892
Nucleotides	1909	1618	1840	2361
Bonds (RMSD)				
Length (A)	0.003	0.003	0.008	0.003
Angles (°)	0.684	0.687	0.675	0.676
Validation				
MolProbity score	1.65	1.90	1.52	1.66
Clash score	6.81	8.27	5.40	6.62
Ramachandran plot (%)				
Outliers	0.01	0.10	0.00	0.01
Allowed	3.99	5.30	3.52	3.14
Favored	95.99	94.60	96.48	96.85
Rotamer outliers (%)	0.76	1.28	0.45	1.39
Model vs. Data				
CC (mask)	0.69	0.61	0.76	0.67
CC (box)	0.77	0.81	0.83	0.77
CC (peaks)	0.62	0.59	0.70	0.63
CC (volume)	0.68	0.61	0.75	0.67

	Nog1TAP_L25-A	Nog1TAP_L25-B	Nog1TAP_L34-A	Nog1TAP_L34-B
Data collection				
Voltage (kV)	300	300	300	300
Pixel size (A)	1.06	1.06	1.06	1.06
Electron exposure (e(A^2)	86.09	86.09	88	88
Fractions	40	40	40	40
Exposure time	5.16	5.16	5.16	5.16
wwPDB accession code	7OHP	7OHW	7OHX	7OHY
EMDB accession code	EMD-12904	EMD-12911	EMD-12912	EMD-12913
EMPIAR accession code	EMPIAR-10774	EMPIAR-10774	EMPIAR-10775	EMPIAR-10775
Map resolution estimates (half m				
Relion (user mask)	3.9	3.5	3.2	3.9
Phenix (autom. mask)	4.0	3.6	3.2	4.0
Model composition				
starting model	6em1	6em1	6em1	6em1
Chains	35	41	39	28
Protein Residues	5290	6824	6076	4067
Nucleotides	1781	1853	1774	1837
Bonds (RMSD)				
Length (A)	0.005	0.003	0.005	0.002
Angles (°)	0.706	0.680	0.705	0.633
Validation				
MolProbity score	1.73	1.61	1.61	1.70
Clash score	7.60	6.15	6.57	7.40
Ramachandran plot (%)				
Outliers	0.16	0.06	0.00	0.03
Allowed	4.32	3.92	3.66	4.16
Favored	95.52	96.02	96.34	95.82
Rotamer outliers (%)	1.01	0.66	0.81	0.61
Model vs. Data				
CC (mask)	0.64	0.75	0.67	0.72
CC (box)	0.80	0.81	0.79	0.82
CC (peaks)	0.58	0.68	0.61	0.68
CC (volume)	0.63	0.75	0.66	0.72
