# Supplementary material for: Analysis of subunit folding contribution of three yeast large ribosomal subunit proteins required for stabilisation and processing of intermediate nuclear rRNA precursors
Source: PLoS One. 2021 Nov 23;16(11):e0252497. doi: 10.1371/journal.pone.0252497 (PMC8610266; doi:10.1371/journal.pone.0252497)

Nog1TAP-A

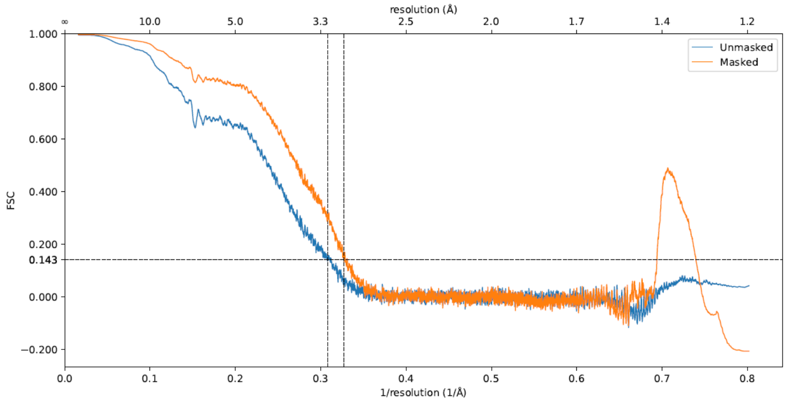

Nog1TAP-B

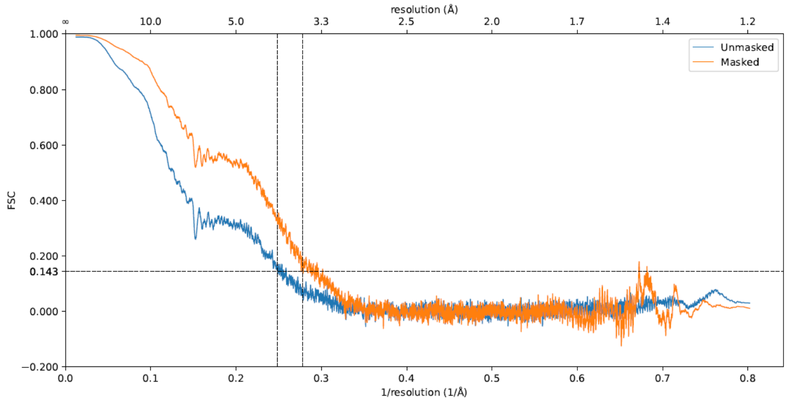

Nog1TAP-C

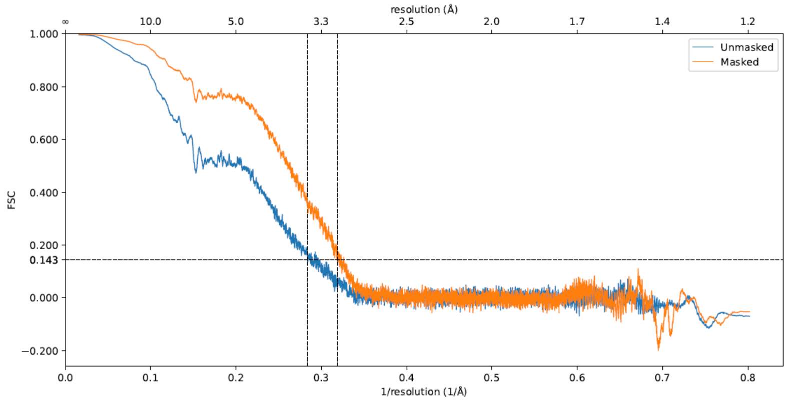

Nog1TAP-E

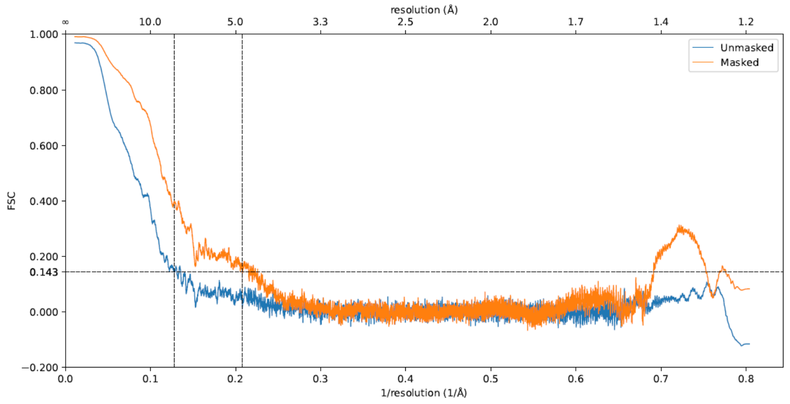

Nog1TAP-F

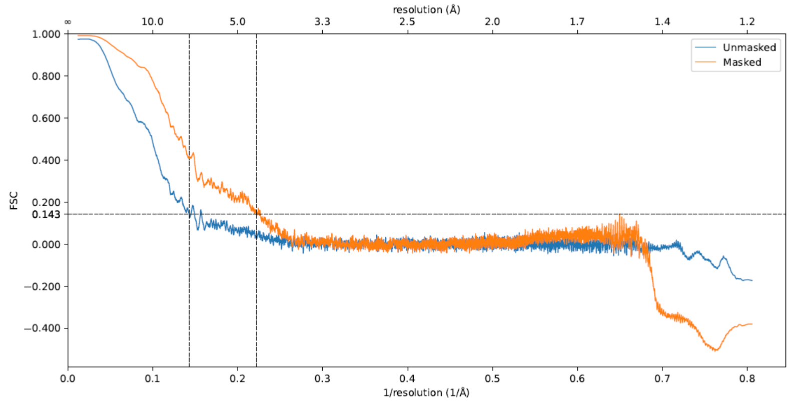

Nog1TAP_L2-A

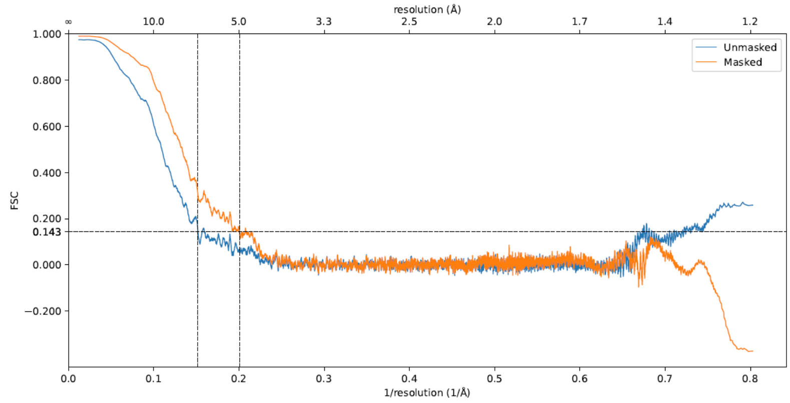

Nog1TAP_L2-B

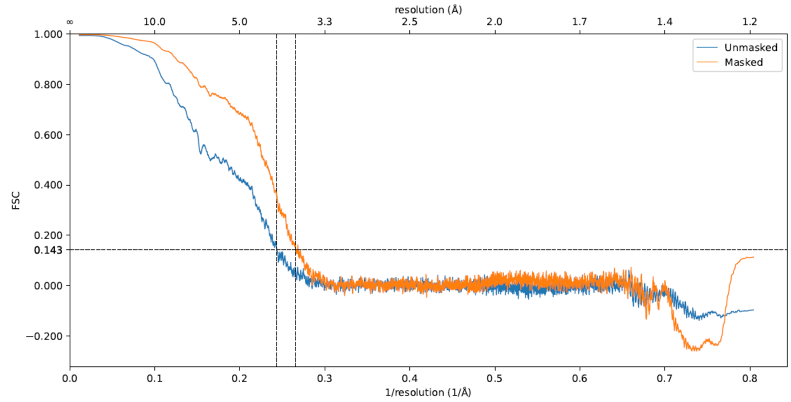

Nog1TAP_L2-C

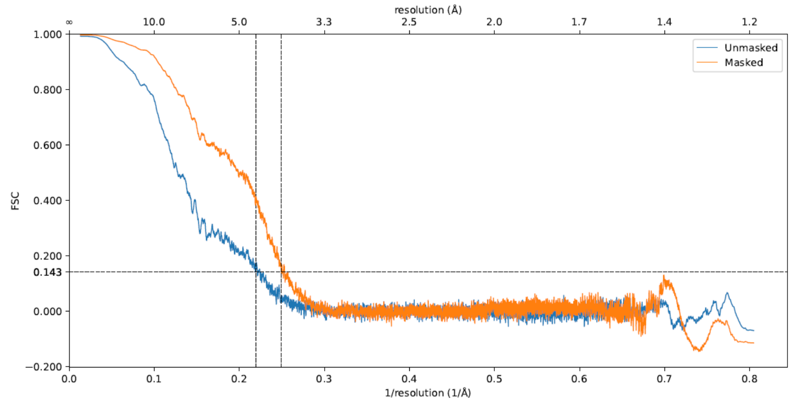

Nog1TAP_L25-A

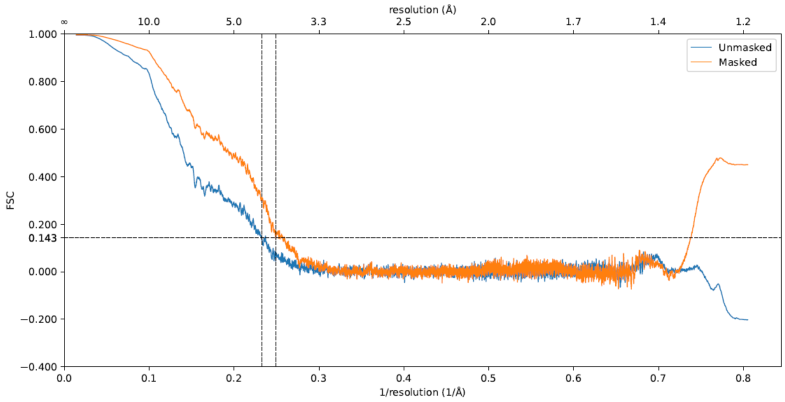

Nog1TAP_L25-B

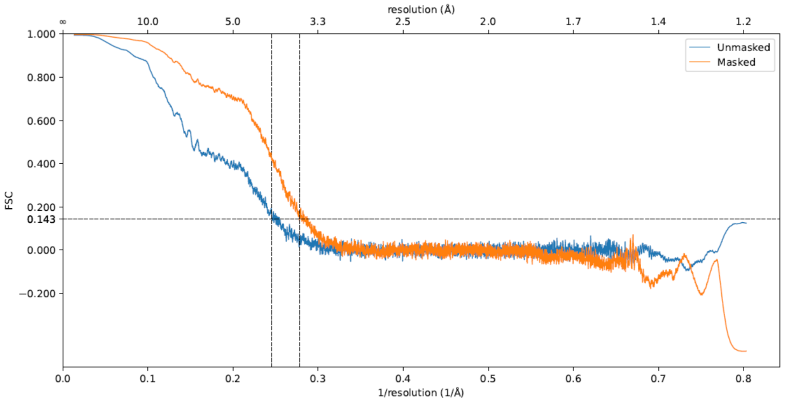

Nog1TAP_L34-A

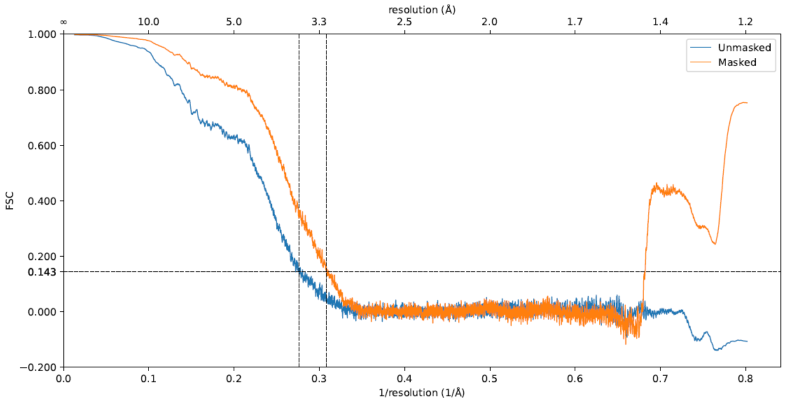

Nog1TAP_L34-B

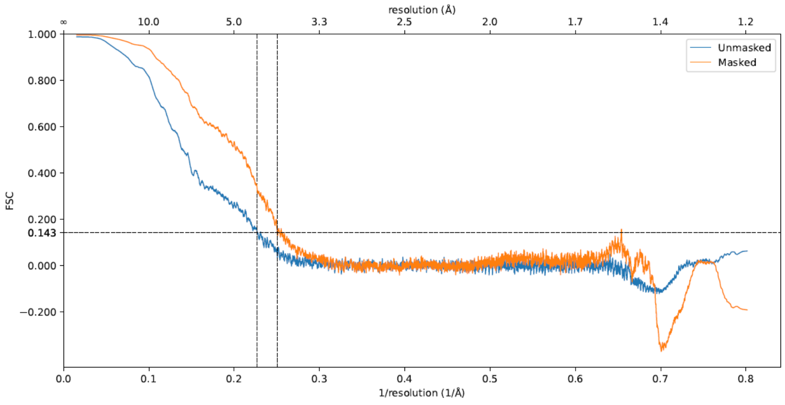

Supplement: S4 Appendix — (PDF) [file pone.0252497.s004.pdf]
