# Supplementary material for: Analysis of subunit folding contribution of three yeast large ribosomal subunit proteins required for stabilisation and processing of intermediate nuclear rRNA precursors
Source: PLoS One. 2021 Nov 23;16(11):e0252497. doi: 10.1371/journal.pone.0252497 (PMC8610266; doi:10.1371/journal.pone.0252497)
Supplement: S5 Appendix — White numbers in 3D classification views indicate particle counts in the respective classes. Data processing schemes are shown for Nog1-TAP associated particles from strain Y1877 in (A), strain Y1921 in (B), strain Y1816 in (C) and Y2907 in (D). All shown intermediate and final density maps obtained by Relion’s 3D-Autorefine procedure are represented using the same dimensions and orientation. (PDF) [file pone.0252497.s005.pdf]

A) Nog1TAP

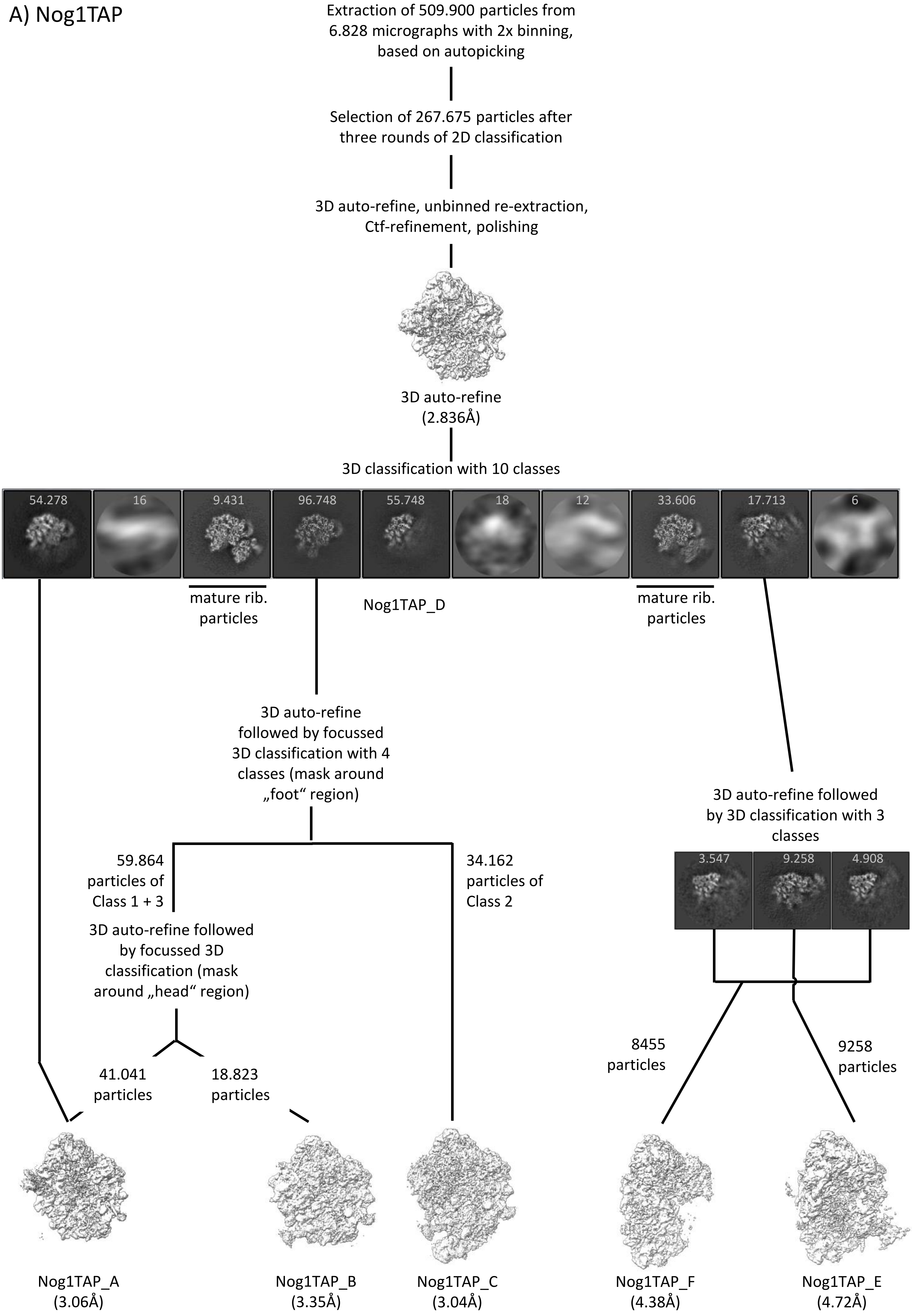

Extraction of 509.900 particles from
6.828 micrographs with 2x binning,
based on autopicking
Selection of 267.675 particles after
three rounds of 2D classification
3D auto-refine, unbinned re-extraction,
Ctf-refinement, polishing
3D auto-refine
(2.836Å)
3D classification with 10 classes
54.278
16
9.431
96.748
55.748
18
12
33.606
17.713
6
mature rib.
particles
Nog1TAP_D
mature rib.
particles
3D auto-refine
followed by focussed
3D classification with 4
classes (mask around
„foot“ region)
59.864
particles of
Class 1 + 3
34.162
particles of
Class 2
3D auto-refine followed
by focussed 3D
classification (mask
around „head“ region)
41.041
particles
18.823
particles
3D auto-refine followed
by 3D classification with 3
classes
3.547
9.258
4.908
8455
particles
9258
particles
Nog1TAP_A
(3.06Å)
Nog1TAP_B
(3.35Å)
Nog1TAP_C
(3.04Å)
Nog1TAP_F
(4.38Å)
Nog1TAP_E
(4.72Å)

## B) Nog1TAP_L2

Extraction of 258.042 particles with 2x binning, based on autopicking

Selection of 98.109 particles with better then 5 A resolution and after two rounds of 2D classification and one round of 3D classification

3D auto-refine, unbinned re-extraction, Ctf-refinement, polishing

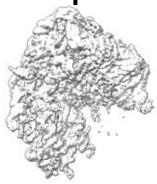

3D auto-refine
(3.57Å)

3D classification with 6 classes

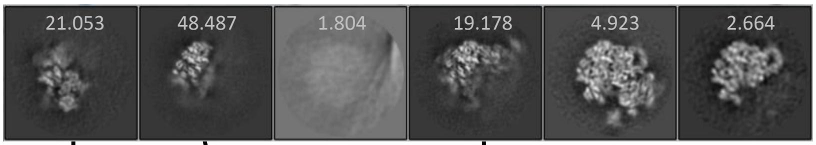


mature ribosomal particles

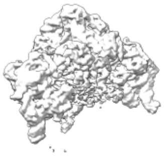

3D auto-refine
Nog1TAP_L2-A
(4.67Å)

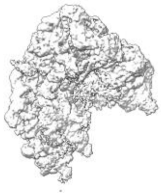

3D auto-refine
Nog1TAP_L2-B
(3.66Å)

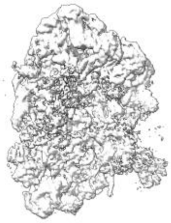

3D auto-refine
Nog1TAP_L2-C
(3.90Å)

C) Nog1TAP_L25

Extraction of 384.469 particles with 2x binning, based on autopicking

Selection of 123.971 particles based on one round of 2D and another round of 2D classification on  particles aligned by 3D auto-refinement

3D auto-refine, unbinned re-extraction, Ctf-refinement, polishing

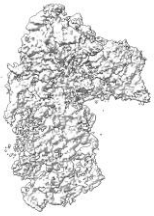

3D auto-refine
(3.4Å)

3D classification with 4 classes

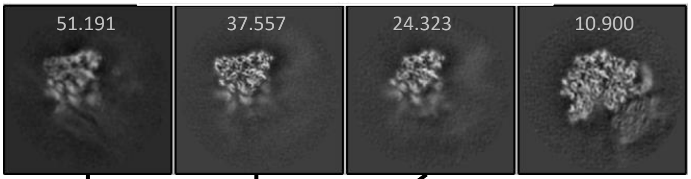


mature rib. particles

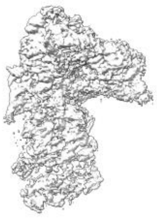

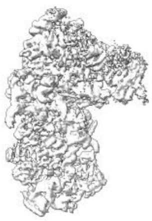

3D auto-refine
Nog1TAP_L25-A
(3.86Å)

3D auto-refine
Nog1TAP_L25-B
(3.48Å)

D) Nog1TAP_L34

Extraction of 244.299 particles with 2x binning, based on autopicking

Selection of 178.955 particles based on one round of 2D classification

Selection of 166.873 candidate pre-60S particles based on one round of 3D classification

3D auto-refine, unbinned re-extraction, Ctf-refinement, polishing

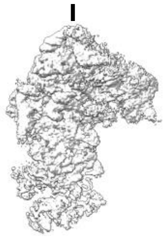

3D auto-refine
(3.2Å)

3D classification with 12 classes

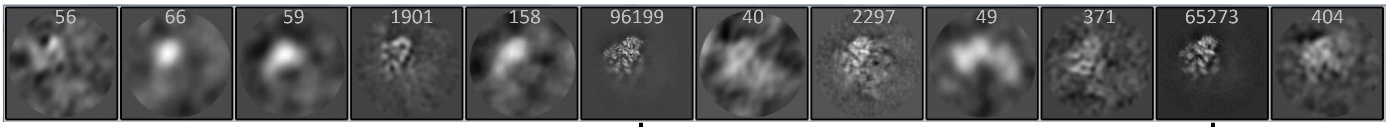


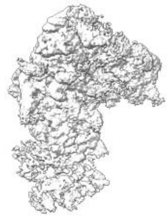

3D auto-refine
(3.17Å)

3D classification with 6 classes

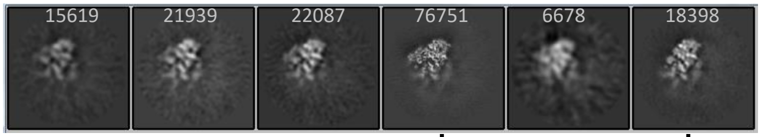


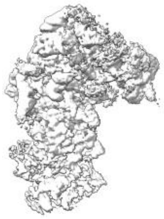

3D auto-refine
Nog1TAP_L34-A
(3.3Å)

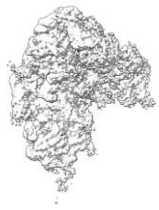

3D auto-refine
Nog1TAP_L34-B
(3.9Å)
