# Supplementary material for: Analysis of subunit folding contribution of three yeast large ribosomal subunit proteins required for stabilisation and processing of intermediate nuclear rRNA precursors
Source: PLoS One. 2021 Nov 23;16(11):e0252497. doi: 10.1371/journal.pone.0252497 (PMC8610266; doi:10.1371/journal.pone.0252497)
Supplement: S8 Appendix — (PDF) [file pone.0252497.s008.pdf]

Fig 1A

27S pre-rRNA signals	
rpL2	0,938749
wt	1
rpL34	2,020892
rpL25	1,009911
rpL2	0,919743
rpL21	0,481912

7S pre-rRNA signals	
rpL2	1,359637
wt	1
rpL34	0,224861
rpL25	0,059801
rpL2	1,119824
rpL21	0,73662

**Ratio 27S pre-rRNA : 7S pre-rRNA**	
**rpL2**	**0,690441**
**wt**	**1**
**rpL34**	**8,987294**
**rpL25**	**16,88775**
**rpL2**	**0,821329**
**rpL21**	**0,654222**

Fig 1B

27S pre-rRNA signals	
rpL2	0,900751
wt	1
rpL34	1,926594
rpL25	0,863335
rpL2	0,699031
rpL21	0,411469

25+25.5S (pre-)rRNA signals	
rpL2	0,241589
wt	1
rpL34	0,1331
rpL25	0,122236
rpL2	0,247076
rpL21	0,661431

**Ratio 27S pre-rRNA : 25+25.5S (pre-)rRNA**	
**rpL2**	**3,728442**
**wt**	**1**
**rpL34**	**14,47477**
**rpL25**	**7,062828**
**rpL2**	**2,829216**
**rpL21**	**0,622089**

Fig 1C

7S pre-rRNA signals	
rpL2	2,361879
wt	1
rpL34	0,337738
rpL25	0,060795
rpL2	1,459231
rpL21	0,9668

5.8S rRNA signals	
rpL2	0,302398
wt	1
rpL34	0,141784
rpL25	0,266287
rpL2	0,208016
rpL21	0,945915

**Ratio 7S pre-rRNA : 5.8S rRNA**	
**rpL2**	**7,81049**
**wt**	**1**
**rpL34**	**2,382062**
**rpL25**	**0,228306**
**rpL2**	**7,014986**
**rpL21**	**1,02208**

All signals were normalized to the respective level of control cells („wt“)
