# Supplementary material for: Analysis of subunit folding contribution of three yeast large ribosomal subunit proteins required for stabilisation and processing of intermediate nuclear rRNA precursors
Source: PLoS One. 2021 Nov 23;16(11):e0252497. doi: 10.1371/journal.pone.0252497 (PMC8610266; doi:10.1371/journal.pone.0252497)
Supplement: S1 Raw images — (PDF) [file pone.0252497.s015.pdf]

Blot Exposition for Fig 1A,
Probe O210,
Image captured with
Typhoon imager FLA9500

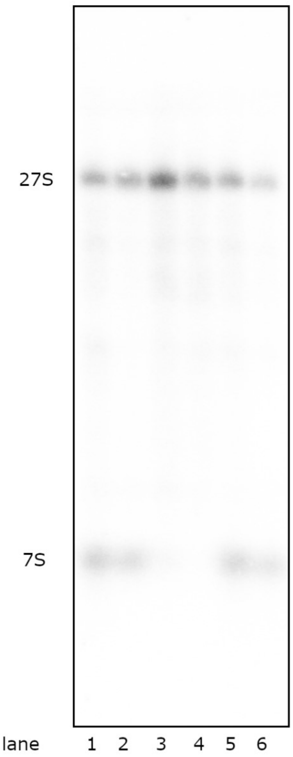

Blot Exposition for Fig 1B,
Probe O212,
Image captured with
Typhoon imager FLA9500

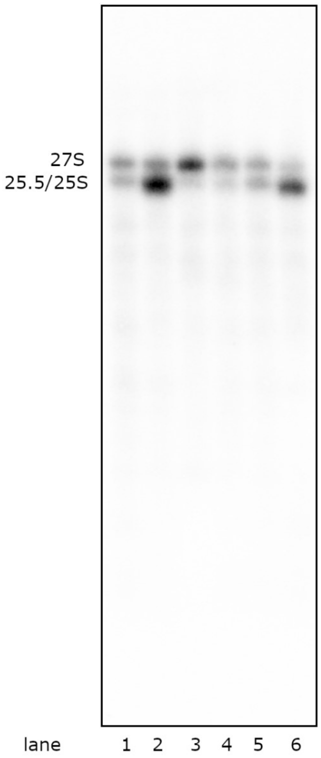

Blot Exposition for Fig 1C,
Probe O209,
Image captured with
Typhoon imager FLA9500

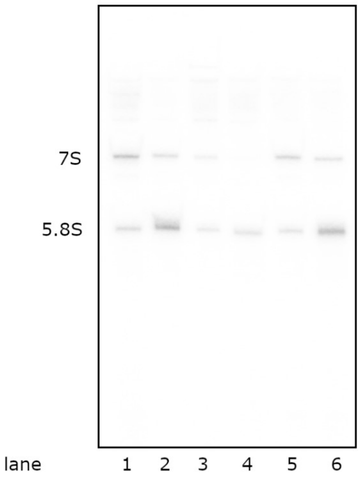

For all expositions RNA from Nog1-TAP purified particles was analyzed by Northern blotting.
In lane 1 rpL2 (Y1921) was depleted,
in lane 2 no protein (Y1877),
in lane 3 rpL34 (Y2907),
in lane 4 rpL25 (Y1816),
in lane 5 rpL2 (Y1921)
and in lane 6 rpL21 (Y1813). All lanes were used in the final Figures.
