# Supplementary material for: Analysis of subunit folding contribution of three yeast large ribosomal subunit proteins required for stabilisation and processing of intermediate nuclear rRNA precursors
Source: PLoS One. 2021 Nov 23;16(11):e0252497. doi: 10.1371/journal.pone.0252497 (PMC8610266; doi:10.1371/journal.pone.0252497)
Supplement: Attachment — Submitted filename: ResponseToReviewers.docx [file pone.0252497.s016.docx]

Dear Dr. Preiss, dear reviewers,

We thank you again for taking the time to inspect the manuscript and for the constructive suggestions to improve its quality.

Reviewer 1 raises the point that the labelling of the figures could still be improved (see below). We agree with reviewer 1 that parts of the text describing changes in the protein composition between different pre-ribosomal populations cannot be easily followed by using the visualization of the structure models shown in Figures 2-6. Due to the large number (twelve) of the described complex models these provide primarily an overview on the general observed rRNA fold from two defined orientations. It is often very challenging to point here on one specific protein out of the easily more than 40 protein components, with many of them embedded into the RNA and intertwined with each other. Besides providing the readers with all pdb models and density maps (currently all with status “hold for publication” in www.pdb.org), the 2D-Maps in S1 Appendix were thought to enable an easier access to the 3D-structure data by showing for each of the twelve models the protein composition, the modeled RNA helices and a simplified 2D-interaction network between them. To follow, as suggested by reviewer 1, the changes in protein compositions also in 3D-representations of the structure models we created for the new revised version an additional supplementary Figure (S9 Appendix). Here, the structure models are visualized in 14 snapshots each with optimized orientation, coloring and zoom level to highlight specific observations raised in the text. We changed the manuscript accordingly, in which we refer now to this additional Figure at several points. We refer now also at a few more points to S1 Appendix with its 2D summary of the structure models. In addition, we corrected a wrong reference on page 5 (line 254) to 5S rRNA helices 5 and 6, now referring to 5S rRNA helices 4 and 5.

We think that, thanks to the reviewer’s suggestions, the quality of the manuscript could be thereby again improved and hope that it is now acceptable for publication in Plos One.

Reviewer 1 suggestions:

“The added labels as well as the movies have improved the manuscript. Still, many parts of the text remain difficult to follow as the Figures are still not sufficiently labelled.

Here are some examples:

"In state Nog1TAP-E (see Fig 2B, Fig 3B and Figure F in S1 Appendix) a group of four factors (Rpf1, Nsa1, Rrp1, Mak16) was possibly released from the subunit. In contrast to state Nog1TAP-F, these could not anymore be detected bound to the rRNA expansion segment ES7 and LSU rRNA domains I and II at the subunit solvent surface."

I tried to identify these factors in the respective regions, However, I was not able to find these proteins supposed to be missing in State E compared to state F. It would be helpful to have e.g.arrows in State E pointing at the positions where proteins have left, or alternatively arrows in State F indicating the proteins that are leaving during the transition from F to E.

"Otherwise, a hallmark of state Nog1TAP-E was the appearance of density unambiguously attributable to LSU rRNA domain III and many of the r-proteins associated with it. Among them were rpL25 and rpL34,…"

While rpL25 is labelled in Nog1-TAP-E particles, rpL34 is not (it is only labelled in later particles), therefore it is difficult to follow the descriptions in the text.

"Several factors (Ytm1, Erb1, Noc3, Ebp2, Brx1, Spb1, Nop16) previously associated with LSU rRNA domains I, II and III were not anymore observed and thus possibly released or with flexible orientation. Various other factors (Rrs1, Rpf2, Rsa4, Nog2, Nop53, Cgr1) and r-proteins (rpL2, rpL43, rpL5, rpL11, rpL21) newly appeared in Nog1TAP-C in association with the emerging rRNA domains."

Same as before – without labeling, it is difficult to follow this text.

I'm aware that the main point of this paper is not the wild-type particles (where much is already known) but changes observed in the particles after ribosomal protein depletion. Still, I believe it is important to first understand the maturation steps of the wild-type particles (which is only possible with better labeling), in order to be able to better compare to the alterations in ribosomal protein-depleted particles.”

We thank reviewer 1 for his comments on the revised version of the manuscript. As described above, to provide a clean way to follow the changes in protein compositions in wildtype cells by 3D structure model representations we added now supplementary Appendix 9 to the manuscript. Here, 14 snapshots of the structure models are shown with optimized orientation, zoom level and specific coloring of protein groups of interest to visualize all the above mentioned and several other observations described in the text.
